# The pathogenicity of vancomycin-resistant *Enterococcus faecalis* to colon cancer cells

**DOI:** 10.1186/s12879-024-09133-2

**Published:** 2024-02-20

**Authors:** Li Zhang, Mingxia Deng, Jing Liu, Jiajie Zhang, Fangyu Wang, Wei Yu

**Affiliations:** 1 Department of Gastroenterology and Hepatology, Jinling Hospital, Affiliated Hospital of Medical School, Nanjing University, Nanjing, Jiangsu China; 2 State Key Laboratory for Diagnosis and Treatment of Infectious Diseases, National Clinical Research Center for Infectious Diseases, Collaborative Innovation Center for Diagnosis and Treatment of Infectious Diseases, The First Affiliated Hospital, Zhejiang University School of Medicine, Hangzhou, China; 3 Department of Infectious Diseases, Zhejiang Provincial People’s Hospital, People’s Hospital of Hangzhou Medical College, Hangzhou, China

**Keywords:** *Enterococcus faecalis*, Colorectal cancer, IL-8, VEGFA, PI3K/AKT/mTOR signaling pathway

## Abstract

**Background:**

The aim of this study was to investigate the pathogenicity of vancomycin-resistant *Enterococcus faecalis* (VREs) to human colon cells in vitro.

**Methods:**

Three *E. faecalis* isolates (2 VREs and *E. faecalis* ATCC 29212) were cocultured with NCM460, HT-29 and HCT116 cells. Changes in cell morphology and bacterial adhesion were assessed at different time points. Interleukin-8 (IL-8) and vascular endothelial growth factor A (VEGFA) expression were measured via RT-qPCR and enzyme-linked immunosorbent assay (ELISA), respectively. Cell migration and human umbilical vein endothelial cells (HUVECs) tube formation assays were used for angiogenesis studies. The activity of PI3K/AKT/mTOR signaling pathway was measured by Western blotting.

**Results:**

The growth and adhesion of *E. faecalis* at a multiplicity of infection (MOI) of 1:1 were greater than those at a MOI of 100:1(*p* < 0.05). Compared to *E. faecalis* ATCC 29212, VREs showed less invasive effect on NCM460 and HT-29 cells. *E. faecalis* promoted angiogenesis by secreting IL-8 and VEGFA in colon cells, and the cells infected with VREs produced more than those infected with the standard strain (*p* < 0.05). Additionally, the PI3K/AKT/mTOR signaling pathway was activated in *E. faecalis* infected cells, with VREs demonstrating a greater activation compared to *E. faecalis* ATCC 29212 (*p* < 0.05).

**Conclusion:**

VREs contribute to the occurrence and development of CRC by promoting angiogenesis and activating the PI3K/AKT/mTOR signaling pathway.

**Supplementary Information:**

The online version contains supplementary material available at 10.1186/s12879-024-09133-2.

## Introduction

*Enterococcus faecalis* is a facultative anaerobic commensal bacterium of the oral cavity and the gastrointestinal tract [1]. However, in susceptible hosts, *E. faecalis* can cause urinary tract infections, infective endocarditis, or transplant infections [2]. Notably, *E. faecalis* has become one of the most common pathogens that causes hospital-acquired infections [3]. Furthermore, the emergence of the vancomycin-resistant *E. faecalis* (VREs) has limited the choice of treatments and has increasingly become a public health threat for hospitals worldwide [4]. VREs infections may lead to significant mortality [5, 6].

Globally, colorectal cancer (CRC) is the third most diagnosed malignancy and the second leading cause of cancer death [7]. Previous studies have demonstrated that the gut microbiota has an essential role in the initiation and promotion of CRC [8]. An imbalance in the intestinal microbiota can affect or impair the integrity of the intestinal epithelium, resulting in inflammation, tumor formation or metastasis progression. Currently, multiple studies have confirmed that the abundance of *E. faecalis* in the faecal microbiota of CRC patients is significantly greater than that in healthy individuals and patients with intestinal polyps [9–11]. *E. faecalis* can polarize macrophages to produce a bystander effect that causes double-stranded DNA breaks, tetraploidy and chromosomal instability (CIN) in target cells and induces inflammation and CRC in interleukin-10 (IL-10) knockout mice [12]. Additionally, *E. faecalis* can generate reactive oxygen species (ROS) and extracellular superoxide, thereby triggering genomic instability and inducing mutations that lead to the development of tumor [13]. In addition, the metalloprotease produced by *E faecalis*, contributes to the development of chronic intestinal inflammation by impairing epithelial barrier integrity [14, 15]. However, most current studies have mainly focused on its function as "driver bacteria" in the occurrence and development of CRC [16]. With the increasing prevalence of VREs, the pathogenicity and underlying mechanisms of VREs in colonic cells have not been determined, and further research is needed on the other roles of VREs in CRC to explore potential treatment targets. Therefore, we explored the pathogenicity of VREs to colonic cells in different stages in vitro.

## Materials and methods

### Bacterial strains

A total of 2 VREs (4942, 12022) were isolated from clinical specimens for diagnosis and frozen at − 80 ℃ in our laboratory. The details of included isolates were described in our previously published article [17]. *E. faecalis* ATCC 29212 was used as a control standard strain. The isolates were inoculated on Mueller–Hinton II agar (MHA) for 24 h at 37 ℃.

### Cell culture

Two commonly used CRC cell lines (HT-29, HCT116) and one human normal colonic epithelial cell line NCM460 were obtained from the American Type Culture Collection (ATCC, Manassas, VA, USA). HT-29 was highly differentiated, and HCT116 was CRC cell line in situ. The human umbilical vein endothelial cells (HUVECs) were also obtained from ATCC. HT-29 cells were routinely cultured in McCoy’s 5A medium (Gibco, Carlsbad, CA, USA), NCM 460 were grown in RPMI 1640 medium (Gibco, Carlsbad, CA, USA), and the other two cell lines were cultured in Dulbecco’s Modified Eagle’s Medium (DMEM) (Gibco, Carlsbad, CA, USA), both of which contained 10% foetal bovine serum (FBS). All the cells were maintained at 37 ℃ in a humidified 5% CO_2_ incubator.

### Bacterial growth and adhesion assay

An invading bacteria solution with a multiplicity of infection (MOI) of 100:1 or 1:1 was prepared with complete cell culture medium to infect the NCM460 and HT-29 cell lines. The cocultures were incubated at 37 ℃ for 2, 4, 6, 8, 12 and 24 h respectively. The cell morphology was observed using an electron microscope. After incubation, the cell culture medium was recovered by centrifugation at 7000 × g for 6 min, after which the cells were washed twice with sterile phosphate-buffered saline (PBS) (Gibco, Carlsbad, CA, USA). Moreover, non-adhered bacteria were removed by washing with PBS. Cells with adhered bacteria were treated with 250 μl of trypsin–EDTA (Gibco, Carlsbad, CA, USA) followed by the addition of 250 μl of culture medium containing FBS. Serial tenfold dilutions were prepared in PBS for both the cell supernatant and cells and plated onto Mueller–Hinton agar at 37 ℃ for 24 h. The growth and adhesion were expressed as a percentage of the number of bacteria in the supernatant and cells to the initial number of bacteria.

### Quantitative real-time PCR

Total RNA was extracted from cells using commercial kit (Yishan Biotech, Shanghai, China) according to the manufacturer’s instructions. The RNA concentration was measured using NanoDrop One Spectrophotometer (Thermo Scientific, USA). Subsequently, 1 μg of total RNA from each sample was reverse transcribed to synthesize complementary DNA (cDNA) using a cDNA synthesis kit (Takara Bio Inc., Japan). The synthesized cDNA was subsequently subjected to real time-PCR on a StepOnePlus™ real-time PCR instrument (Applied Biosystems, Foster, CA, USA) to evaluate the gene expression of IL-8 and VEGFA. GAPDH was used as an internal control. The sequences of primer used were as follows: IL-8 forward, 5’- ACATACTCCAAACCTTTCCACC-3′ and reverse, 5’-AAAACTTCTCCACAACCCTCTG-3′; GAPDH forward, 5’-CTGGGCTACACTGAGCACC-3′ and reverse, 5’-AAGTGGTCGTTGAGGGCAATG-3′. VEGFA forward, 5’-ATGAACTTTCTGCTGTCTTGG-3′ and reverse, 5’- TCACCGCCTCGGCTTGTCACA-3; Each experiment was performed independently three times. The expression of IL-8 and VEGFA genes was normalized to GAPDH and calculated based on the 2^−△△CT^ method.

### Bacterial conditioned medium

Prepared the invading bacteria solution with the MOI of 100:1 and 1:1 with complete cell culture medium to infect NCM460, HT-29 and HCT116 cell lines. The co-incubated isolates and cells were cultured continuously for 6 h at 37 ℃. The bacteria culture medium was subsequently centrifuged at 6000 × g for 10 min and filtered through a 0.22 mm pore-size filter to obtain the conditioned medium.

### Cell migration assay

Cell Migration Assay Kit (BD Biosciences, NJ, USA) was used to evaluate the migration of HUVECs. Briefly, 250 μl serum-free medium containing 2.0 × 10^4^ HUVECs was added to the upper chamber of an 8-μm pore in a 24-well plate, and 750 μl of conditioned supernatant was added to the lower chamber. Subsequently, the cells were incubated in this system for 24 h at 37 ℃. After removing non-invading cells with cotton swabs, the migrated cells were fixed with 4% paraformaldehyde and stained with 0.1% crystal violet solution (Sigma, St. Louis, MO). Finally, stained cells were counted in at least three randomly selected fields at 100X magnification under a microscope to minimize bias.

### Endothelial tube formation assay

HUVECs (2 × 10^4^) were plated in 96-well plates coated with 50 μl Matrigel (BD Biosciences, Bedford, MA) and cultured in conditioned culture medium for 6 h at 37 °C with 5% CO_2_. Tubules were photographed with a microscope and evaluated by Image-Pro Plus software.

### Enzyme-linked immunosorbent assay (ELISA)

The protein levels of IL-8 and VEGFA in the bacterial conditioned supernatant were measured with ELISA kits (Proteintech Group, Wuhan, China, #KE00006 for IL-8; RayBiotech, Inc. Georgia, GA, USA, #ELH-VEGF-1 for VEGFA) according to the manufacturer’s instructions.

### Western blot analysis

Total protein was extracted using radio immunoprecipitation assay (RIPA) lysis buffer (Beyotime Institute of Biotechnology, Shanghai, China) buffer containing phosphatase and protease inhibitors. The proteins were quantified using bicinchoninic acid (BCA) assay, separated via 10% SDS-PAGE and subsequently transferred to a polyvinylidene fluoride (PVDF) membrane (Beyotime Institute of Biotechnology, Shanghai, China). The membrane was blocked with 5% BSA for 1 h and then according to the molecular weight, the membrane was cut and incubated overnight with the corresponding primary antibody, including anti-Actin mouse antibody (66009–1-Ig, Proteintech Group, 1:5000), anti-PI3K mouse antibody (60225–1-Ig, Proteintech Group, 1:5000), anti-AKT mouse antibody (60203–2-Ig, Proteintech Group, 1:2000), anti-mTOR rabbit antibody (2972S, Cell Signaling Technology, 1:1000), anti-p-PI3K rabbit antibody (AF3242, Affinity Biosciences, 1:1000), anti-p-AKT mouse antibody (66444–1-Ig, Proteintech Group, 1:2000), anti-p-mTOR rabbit antibody (5536 T, Cell signaling Technology, 1:1000). After washing with Tris-buffered saline plus Tween®20 (TBST), the membranes were incubated with secondary antibody (1:5000) for 1 h, and chemiluminescence was used to visualize the protein bands with X-ray film. The intensity was analyzed by Image J software.

### Statistical analysis

All the data analyses in our study were performed using GraphPad Prism 8 (GraphPad, USA). All the experiments were performed in three or more replicates. The data are presented as the mean ± standard deviation (SD). Analysis of variance (ANOVA) was used to compare differences among multiple groups. Comparisons between two groups were performed using Mann–Whitney U-test. *p*-values < 0.05 indicated statistical significance.

## Results

### Cell morphology in bacteria adhesion assay

**Fig. 1:**
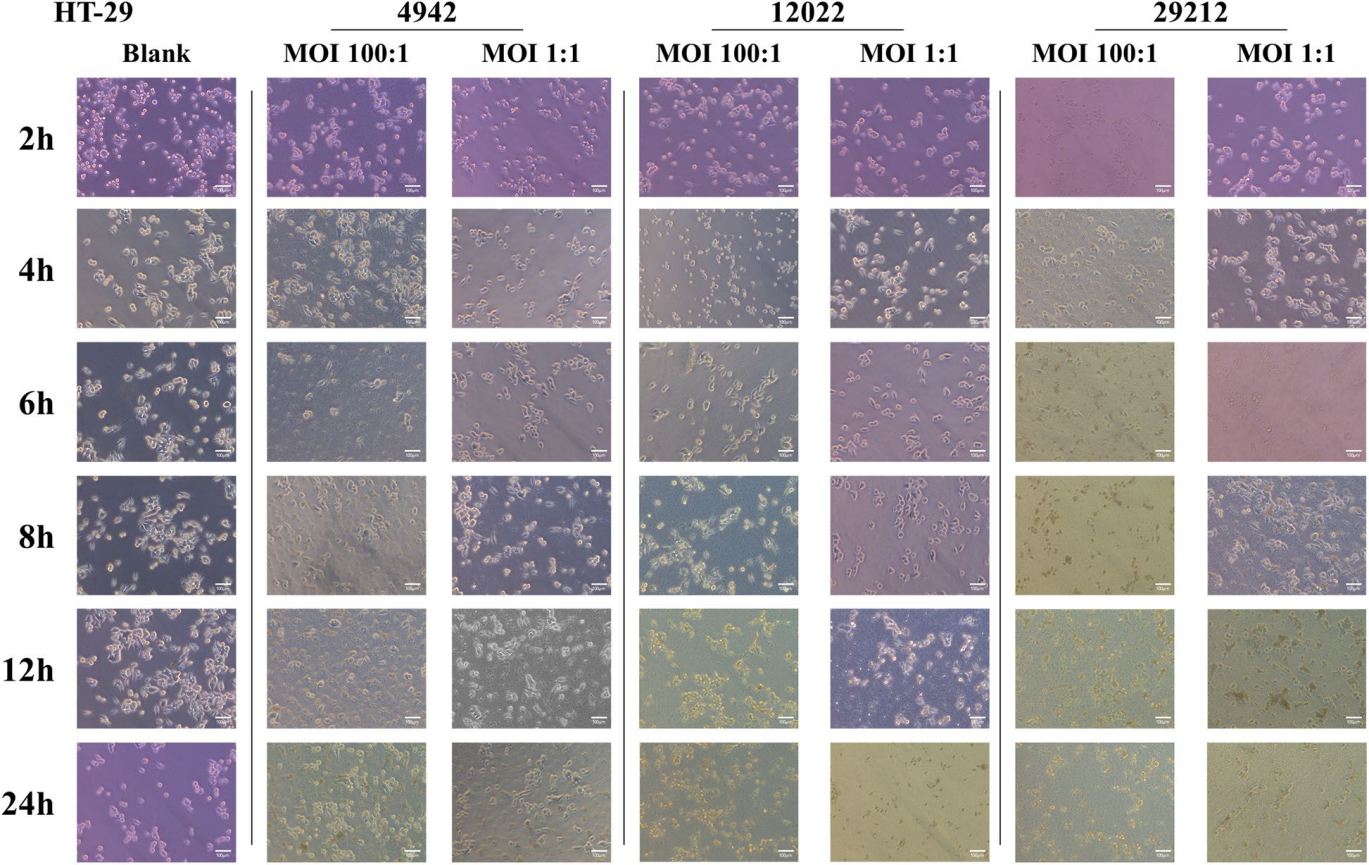
Morphological changes of HT-29 infected with different *Enterococcus faecalis* (VREs: 4942, 12022 and *E. faecalis* ATCC 29212). Scale bar, 100 μm

All the included isolates were suspended at concentrations of 100:1 and 1:1 in cell culture medium. With the extension of coculture time, we observed that the bacteria continuously reproduced, while the cells gradually swelled, ruptured and died. Moreover, compared to *E. faecalis* ATCC 29212, VREs were found to be less invasive to NCM460 and HT-29. Treatment with *E. faecalis* ATCC 29212 resulted in cell death at 6 h, while VREs caused the release of cell secretions at 8 h (Fig. 1 and Supplementary Figure S1).

### Bacterial growth and adhesiveness

**Fig. 2:**
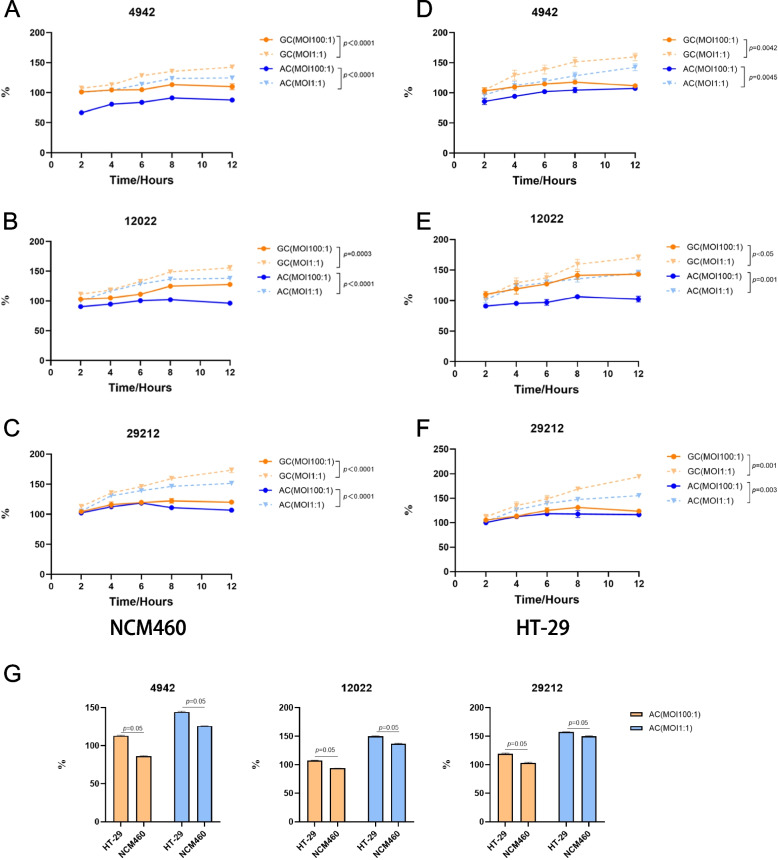
The growth and adhesion assay of two VREs (4942, 12022) and *E. faecalis* ATCC 29212. **A**, **B**, **C**, **D**, **E**, **F** The solid lines indicated multiplicity of infection (MOI) 100:1 and the dotted lines indicated MOI 1:1. The orange line represents the bacterial growth curve (GC) and the blue line represents the adhesion curve (AC). The left panel represents NCM460, and the right panel represents HT-29. **G** The adhesion of *E. faecalis* to two cell lines after coculturing for 12 h. The orange bars represent MOI 100:1 and the blue bars represent MOI 1:1. Results are presented as mean ± SD (*n* = 3)

As shown in Fig. 2(A-F), the proliferation and adhesion of *E. faecalis* were significantly increased at the low MOI. At the time point of 12 h, the proliferation ability and the adhesion capacity of *E. faecalis* under the low MOI were more than 1.3 times that of high MOI (*p* < 0.05).

In addition, after coculture for 12 h, the adhesion of *E. faecalis* to the CRC cell line HT-29 was stronger than that to the normal colonic epithelial cell NCM460 (Fig. 2G).

**Fig. 3:**
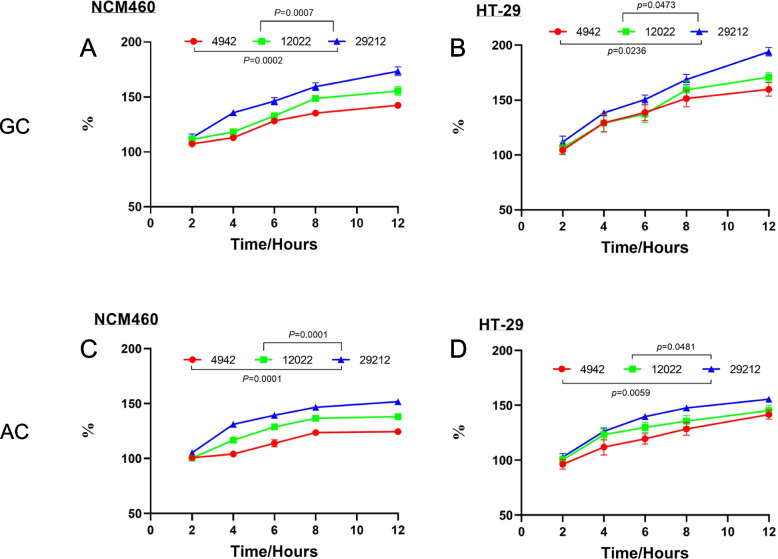
The comparison of growth and adhesion ability between three *E. faecalis* strains at the MOI of 1:1. **A**, **B** The bacterial growth curve. **C**, **D** The bacterial adhesion curve. The red line represents VREs 4942; the green line represents VREs 12022; The blue line represents *E. faecalis* ATCC 29212. Results are presented as mean ± SD (*n* = 3)

Furthermore, we compared the proliferation and adhesion of the three strains at the MOI of 1:1. It was found that *E. faecalis* ATCC 29212 was markedly more abundant than VREs (Fig. 3) (*p* ≤ *0.0481*), which was also consistent with earlier observations regarding the cell death time after infection. And after coculturing for 12 h, the proliferation and adhesion of *E. faecalis* ATCC 29212 were 1.2 times more than VREs.

### VREs promoted the expression of IL-8 and VEGFA in colon cell lines cells

**Fig. 4:**
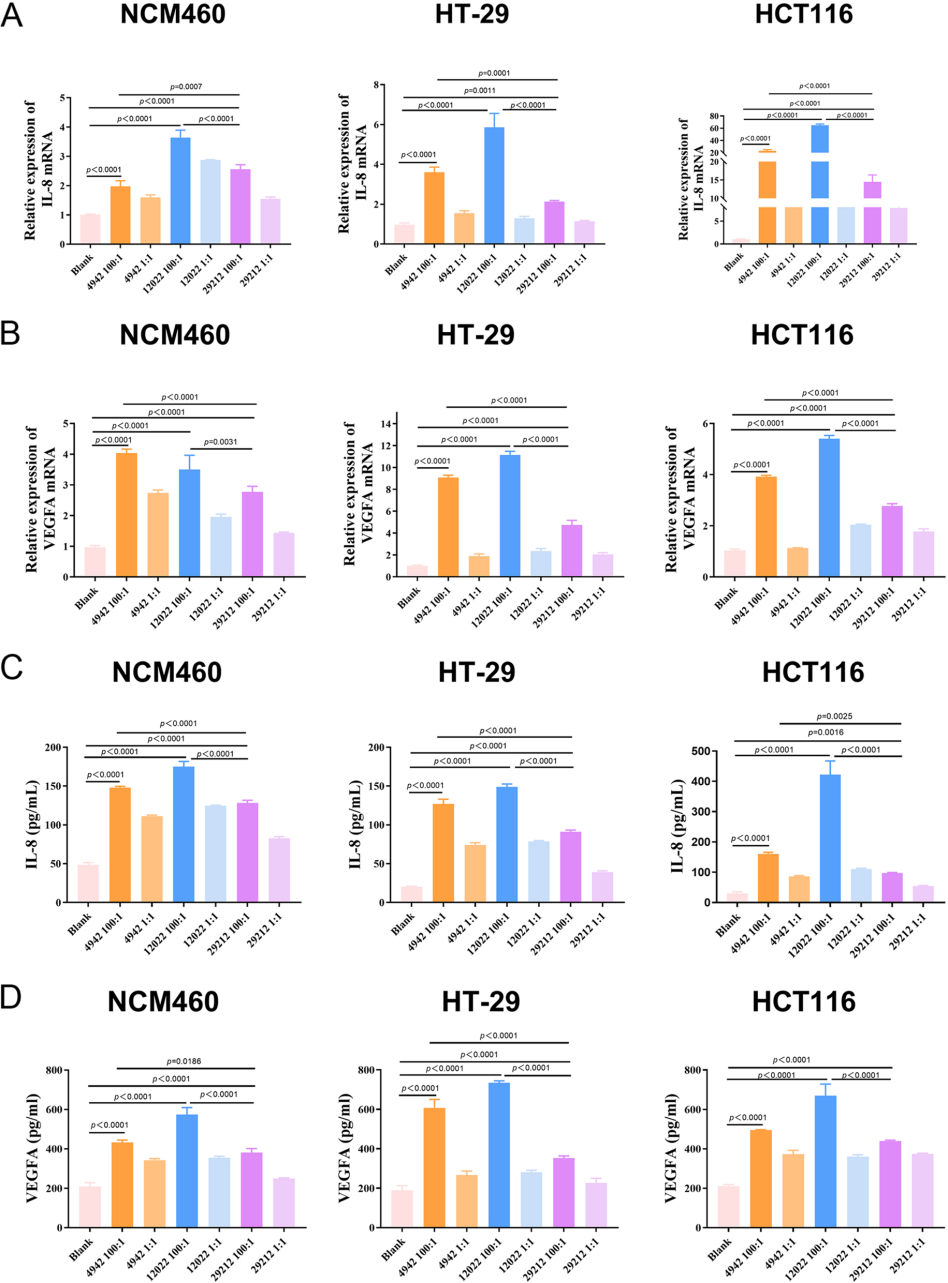
VREs promoted the expression of IL-8 and VEGFA in colonic cell lines. **A**, **B** The expression of IL-8 and VEGFA were detected by RT-qPCR in NCM460, HCT116 and HT-29 cells cocultured with *E. faecalis* (4942, 12022 and *E. faecalis* ATCC 29212). Results are expressed as fold increase of control. **C**, **D** The concentration of IL-8 and VEGFA in the culture supernatant were examined by Elisa. Data are mean ± SD (*n* = 3)

The mRNA expression of IL-8 and VEGFA were significantly upregulated in all the three cells cocultured with *E. faecalis* (Fig. 4A-B). Specifically, the expression of IL-8 and VEGFA in the experimental groups were more than twice that of the control group, with IL-8 exceeding 60 times and VEGFA exceeding 10 times (*p* < 0.0001).

In addition, ELISA assays demonstrated that *E. faecalis* significantly increased the extracellular secretion of IL-8 and VEGFA compared to normal control, and the highest values exceeded 13 times and 3 times, respectively(*p* < 0.0001). Furthermore, cells infected with VREs produced more than twice as much IL-8 and VEGFA as cells infected with *E. faecalis* ATCC 29212(*p* ≤ 0.0186) (Fig. 4C-D).

### VREs promotes angiogenesis in vitro

**Fig. 5:**
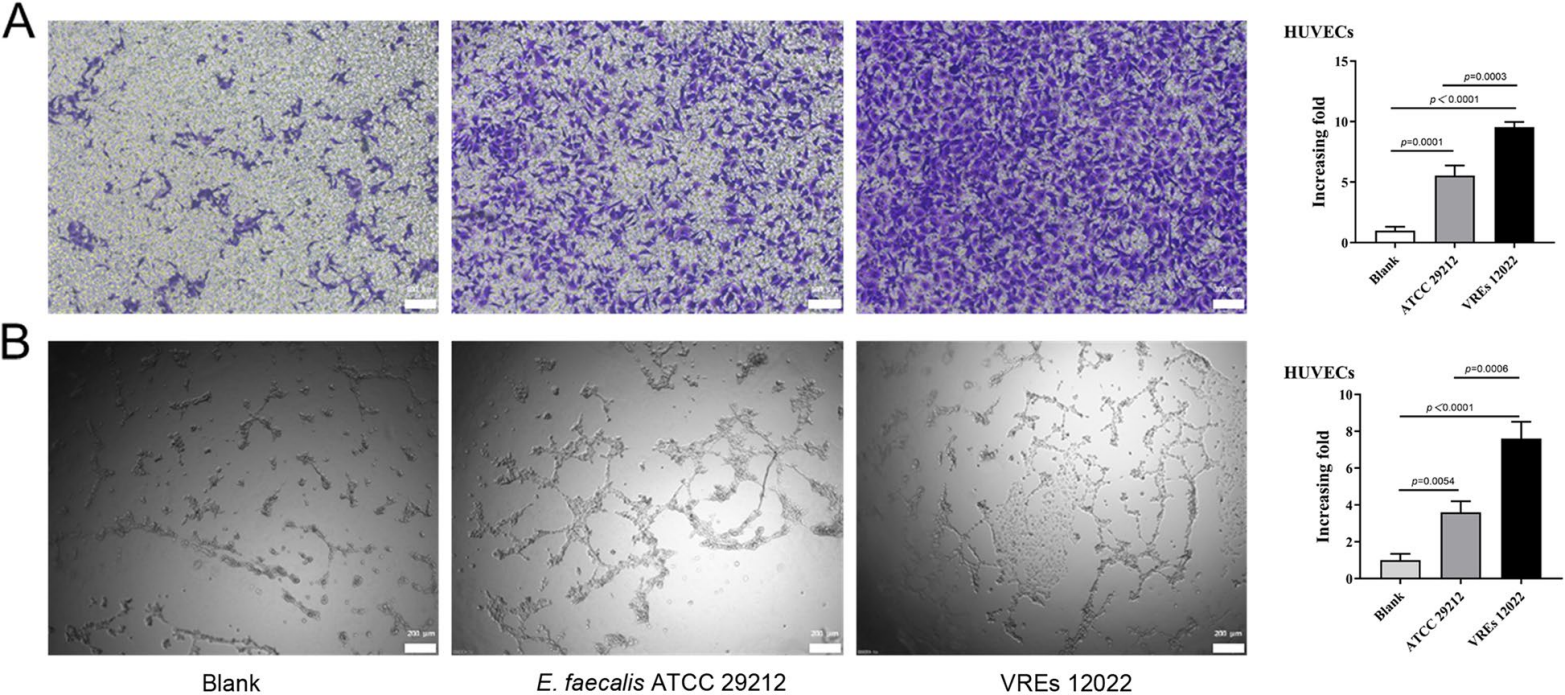
VREs stimulated endothelial cell migration and tube formation. **A** Cell migration in HUVECs were examined by transwell assays after HUVECs were plated and cocultured with the bacterial conditioned medium. One representative image from three reproducible experiments is shown. Scale bar, 100 μm. The increasing folds of migrated HUVEC numbers are shown in the bar graph. **B** HUVECs tube formation were shown in representative images after co-incubating with the bacterial conditioned medium. Scale bar, 200 μm. The increasing folds of tube formation is shown in the bar graph

Endothelial cell migration is critical for angiogenesis. Therefore, we evaluated the potential role of *E. faecalis* on angiogenesis in vitro through the migration and tube formation of HUVECs. As with IL-8 and VEGFA secretion, VREs conditioned medium significantly promoted HUVECs migration(*p* = 0.0003) (Fig. 5A) and tube formation ability of HUVECs (Fig. 5B) (*p* = 0.0006). These results confirmed the important effect of VREs on angiogenesis in HUVECs.

### Activation of the PI3K/AKT/mTOR pathway in cells infected with VREs

**Fig. 6:**
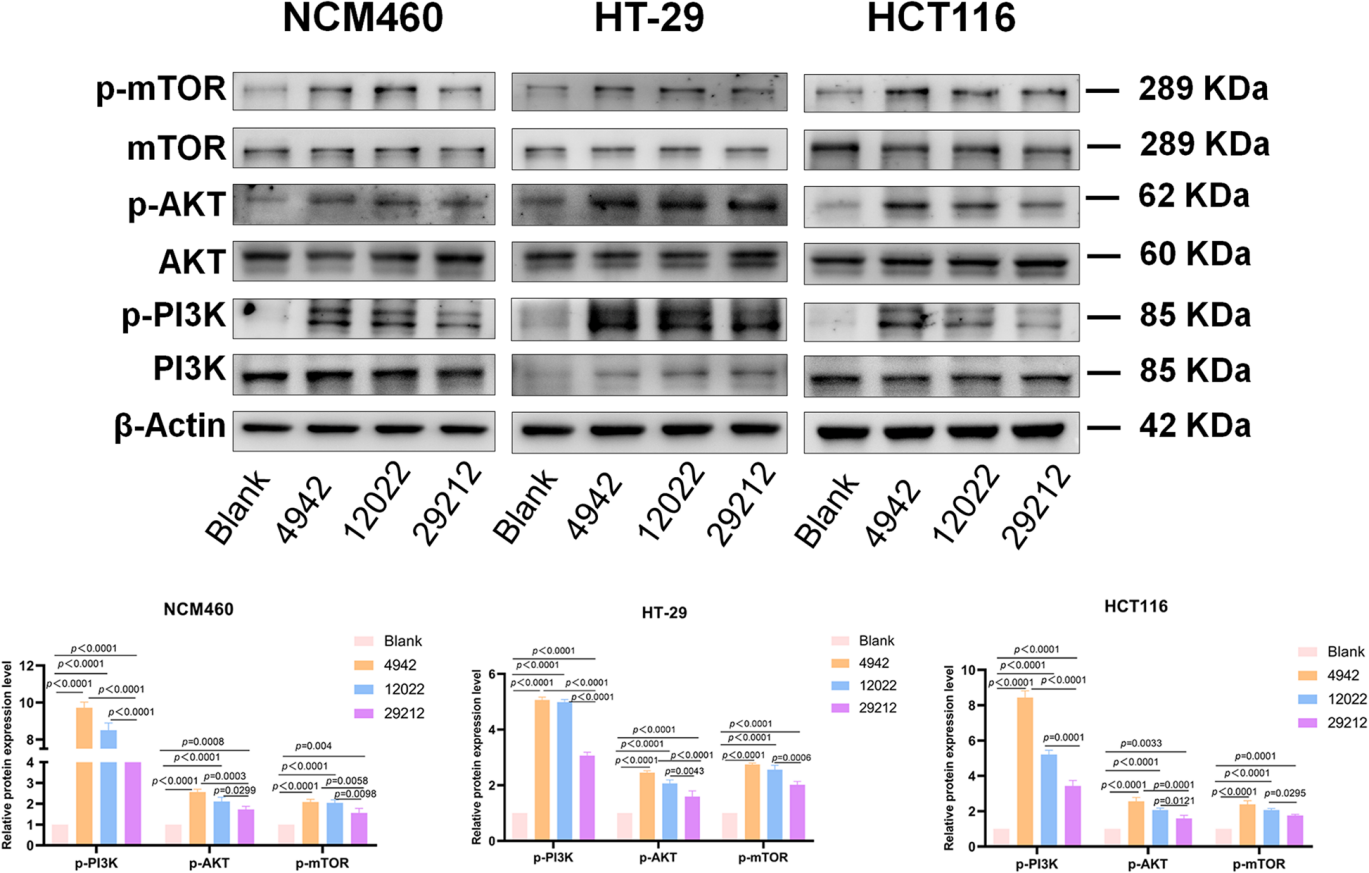
VREs activated the PI3K/AKT/mTOR pathway in cells

Since VREs had a stronger stimulating effect on colon cell lines at the MOI of 100:1, we extracted proteins from infected cells at this concentration. The PI3K/AKT/mTOR signaling pathway was activated in *E. faecalis* infected cells, and consistent with the secretion of IL-8 and VEGFA, the expression level of phosphorylated proteins induced by VREs were more than twice that of *E. faecalis* ATCC 29212 (*p* ≤ 0.0299) (Fig. 6).

The expression of PI3K/AKT/mTOR pathway members were detected by western blot in the three colonic cell lines treated with or without *E. faecalis* (VREs 4942, 12022 and *E. faecalis* ATCC 29212).

## Discussion

In recent years, *E. faecalis* has been shown to cause the occurrence and development of CRC through oxidative stress and chromosomal instability [18]. In this study, *E. faecalis* increased the expression and secretion of IL-8 and VEGFA, and activated the PI3K/AKT/mTOR signaling pathway. In addition, the pathogenicity of VREs was striking.

Our findings showed that *E. faecalis* with low MOI had stronger adhesion to infected cells. This difference may be related to the living space of bacteria. Competition for space is ubiquitous in the ecology of both micro-organisms and macro-organisms [19]. To survive and thrive under a variety of biotic and abiotic pressures, bacteria must communicate, cooperate, and compete with the surrounding community [20, 21]. As previously reported, bacteria can deliver toxins through Type VI secretion systems (T6SS) and contact-dependent-inhibition (CDI) systems to influence competitors and interact antagonistically to obtain additional living space [22–25]. T3SS were identified in VREs. Therefore, *E. faecalis* with low MOI have more space for sustainable growth, which is more conducive to bacterial proliferation and adhesion. In addition, in our study, we demonstrated that *E. faecalis* has different adhesion abilities to different cells. Compared with the normal colon epithelial cell NCM460, *E. faecalis* showed stronger adhesion to colon cancer cell HT-29. The expression of some receptors on the surface of colon cancer cells or changes in cancer cell morphology may be related to increased adhesion of bacteria [26].

Overexpression of the PI3K/AKT/mTOR signaling pathway has been reported in different forms of CRC [27, 28]. The activation of PI3K/AKT/mTOR signaling pathway could promote the production of IL-8 [29, 30]. IL-8 has been demonstrated to be a critical cytokine that is involved in CRC progression. IL-8 can promote CRC metastasis by inducing the epithelial-mesenchymal transition (EMT), inhibiting apoptosis and increasing tumor angiogenesis [31]. Furthermore, an increase in the level of IL-8 is associated with poor prognosis and drug resistance in many cancers [32, 33]. Angiogenesis is an essential process for the growth and proliferation of solid tumors [34], and VEGFA is the main mediator of angiogenesis [35, 36]. Current evidence has shown that VEGFA levels and VEGF receptor activity are associated with poor prognosis in CRC patients [37]. Our results indicated that *E. faecalis* exposure resulted in significant increases in the expression of IL-8 and VEGFA in normal colonic cell and CRC cells. In addition, *E. faecalis* promoted the migration and tube formation of HUVECs in vitro, suggesting that *E. faecalis* may be involved in angiogenesis during the development of colorectal cancer. However, further researches are needed to confirm these findings.

Interestingly, compared with those in the standard strain (*E. faecalis* ATCC 29212), the effects of VREs on the activation of signaling pathway and the secretion of IL-8 and VEGFA in colonic cell lines were significantly greater. However, the invasive effect of VREs was weak in the growth and adhesion experiments. Geraldes et al. [38] suggested that VREs play a leading role in enterococcal infections and considered the primary species in terms of nosocomial infections. The virulence factors of *enterococcus* can be roughly divided into two distinct groups: those that are secreted and those that are present in the surface of bacterial cells [39]. Cytolysin is one of the earliest secreted virulence factors that was identified in enterococci [40], which can damage host cell membranes and facilitate infection. Other types of cell surface protein virulence factors, such as microbial surface components recognizing adhesive matrix molecules (MSCRAMMs), Esp, aggregation substances (Asp1, Asp10 and Asa1) and Pili (EBP, BEE, PGC1-4), which have been found to be important for a range of different bacterial defense mechanisms, including biofilm formation and protection of the host immune system [41–43]. In our previous study, we demonstrated that VREs exhibited high-level resistance but only harbor virulence genes related to cytolysin and the biofilm forming ability of VREs was weaker than that of standard strains [17]. Therefore, due to the fewer cell surface virulence factors of VREs, it is possible that these strains exhibit weaker adhesion to colonic cells, leading to a more lasting and stronger activation in coculture systems.

Although this study is exploratory in nature, there were several limitations. First, the sample sizes of the experimental strains and cells were small. Second, animal experiments were not performed. Further animal studies or clinical trials are warranted to validate these findings.

## Conclusion

In conclusion, our findings showed that VREs may promote the secretion of IL-8 and VEGFA and activate the PI3K/AKT/mTOR signaling pathway, resulting in an angiogenic phenotype that stimulates the progression of CRC. This study advanced our understanding of the pathogenicity of *E. faecalis*, especially drug-resistant strain in CRC. Additional in vivo studies and validation with clinical samples are worth further investigation to explore the molecular mechanism of this microorganism in CRC, in order to expand the relationship between the microbial community and CRC.

### Supplementary Information

**Supplementary Material 1.**

**Supplementary Material 2.**

## Data Availability

The datasets used and/or analysed during the current study available from the corresponding author on reasonable request.
