# Supplementary material for: The pathogenicity of vancomycin-resistant Enterococcus faecalis to colon cancer cells
Source: BMC Infect Dis. 2024 Feb 20;24:230. doi: 10.1186/s12879-024-09133-2 (PMC10880345; doi:10.1186/s12879-024-09133-2)
Supplement: Supplementary file 1 — Supplementary Material 1. [file 12879_2024_9133_MOESM1_ESM.pdf]

Supplementary Figure S1

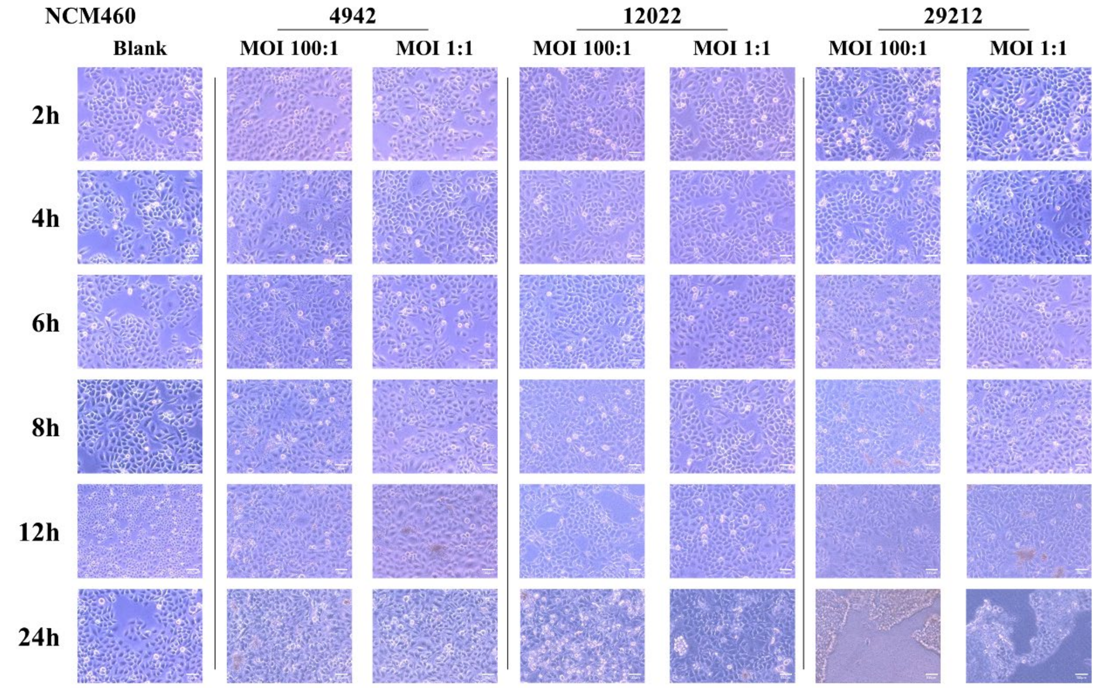

Supplementary Figure S1. Morphological changes of NCM460 infected with different *Enterococcus faecalis* (VREs: 4942, 12022 and ATCC 29212). Scale bar, 100μm.
