# Supplementary material for: The pathogenicity of vancomycin-resistant Enterococcus faecalis to colon cancer cells
Source: BMC Infect Dis. 2024 Feb 20;24:230. doi: 10.1186/s12879-024-09133-2 (PMC10880345; doi:10.1186/s12879-024-09133-2)
Supplement: Supplementary file 2 — Supplementary Material 2. [file 12879_2024_9133_MOESM2_ESM.docx]

**Blots used in the paper:**

**NCM460 Actin (42 KDa)：**


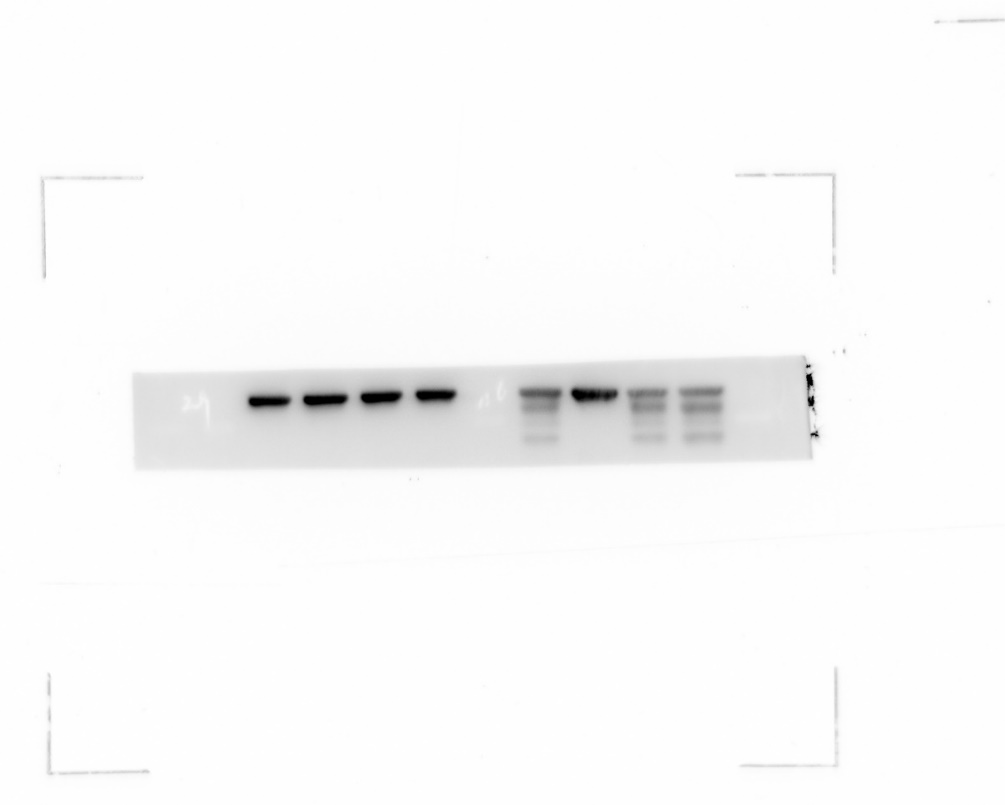


40KDa

**HT-29 Actin (42 KDa)：**


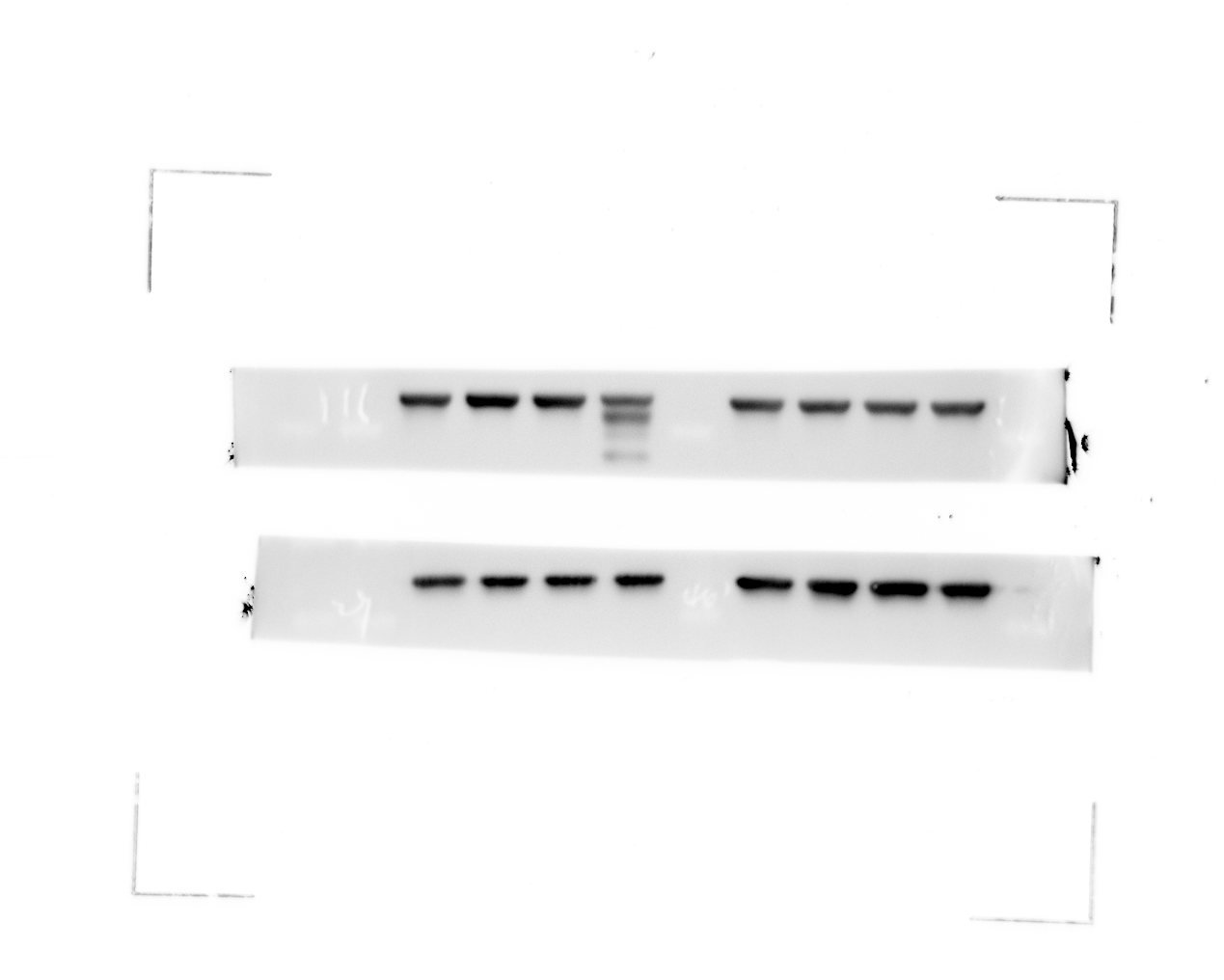


40KDa

**HCT116 Actin (42 KDa)：**


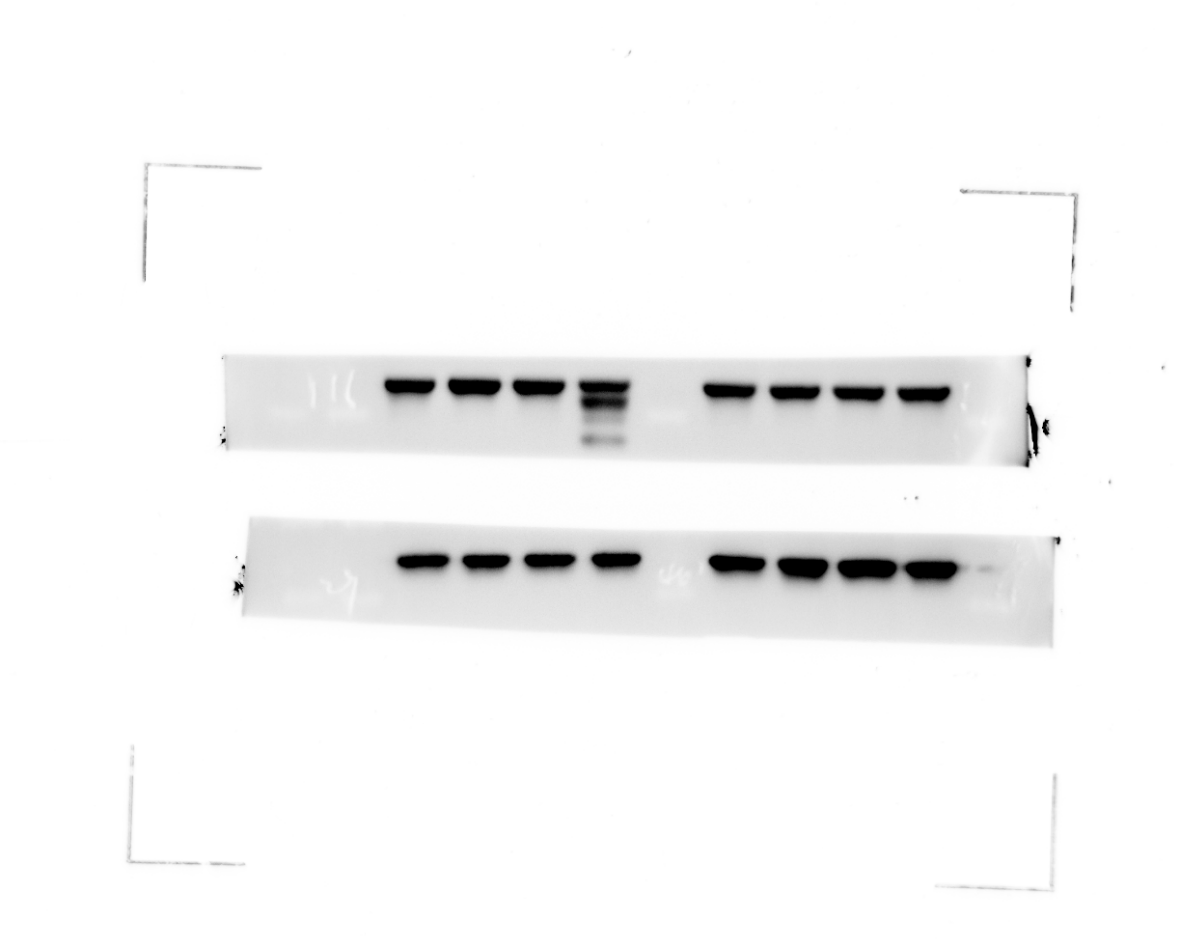


40KDa

**HT-29 AKT (60 KDa):**


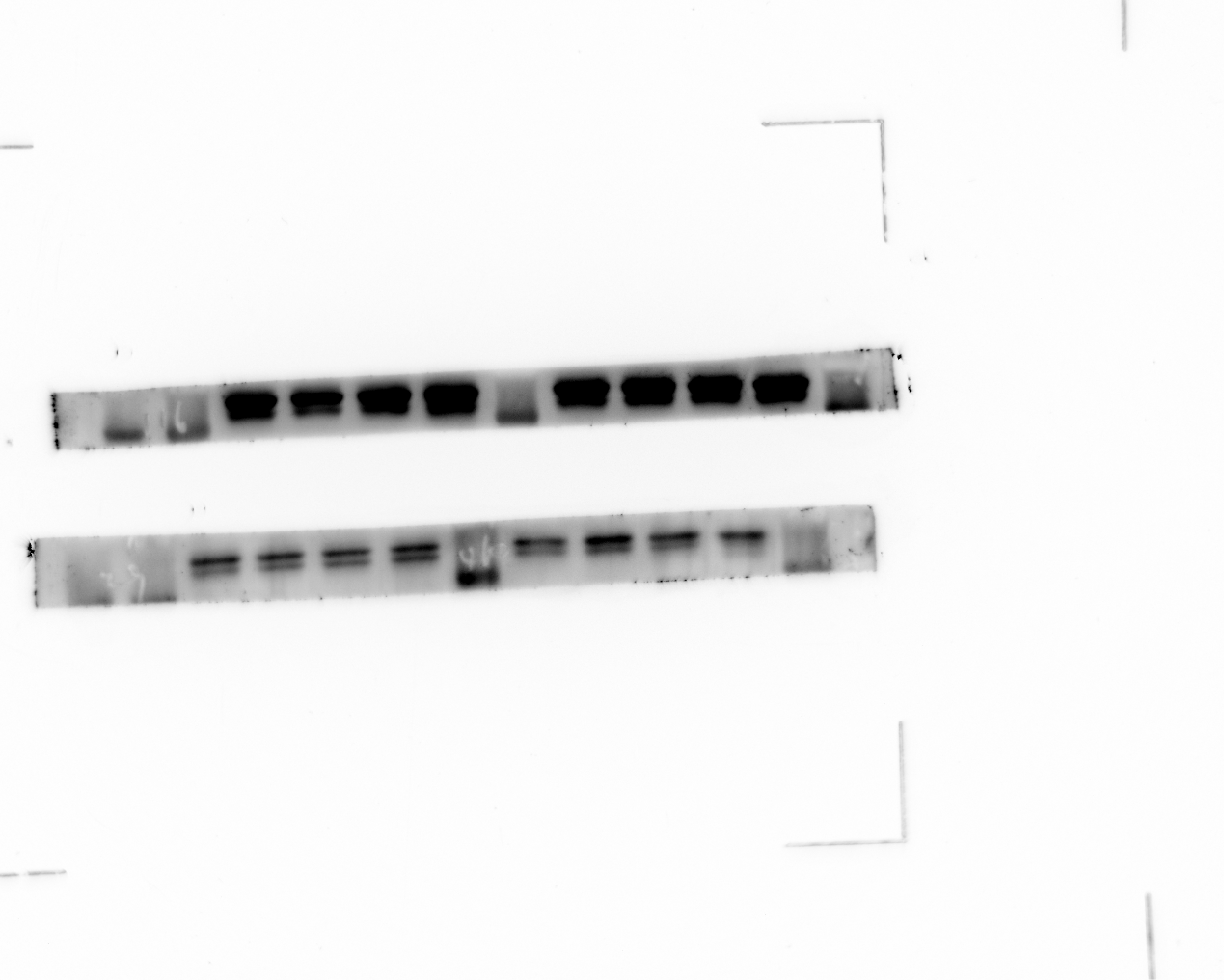


55KDa

**NCM460+HCT116 AKT (60 KDa):**


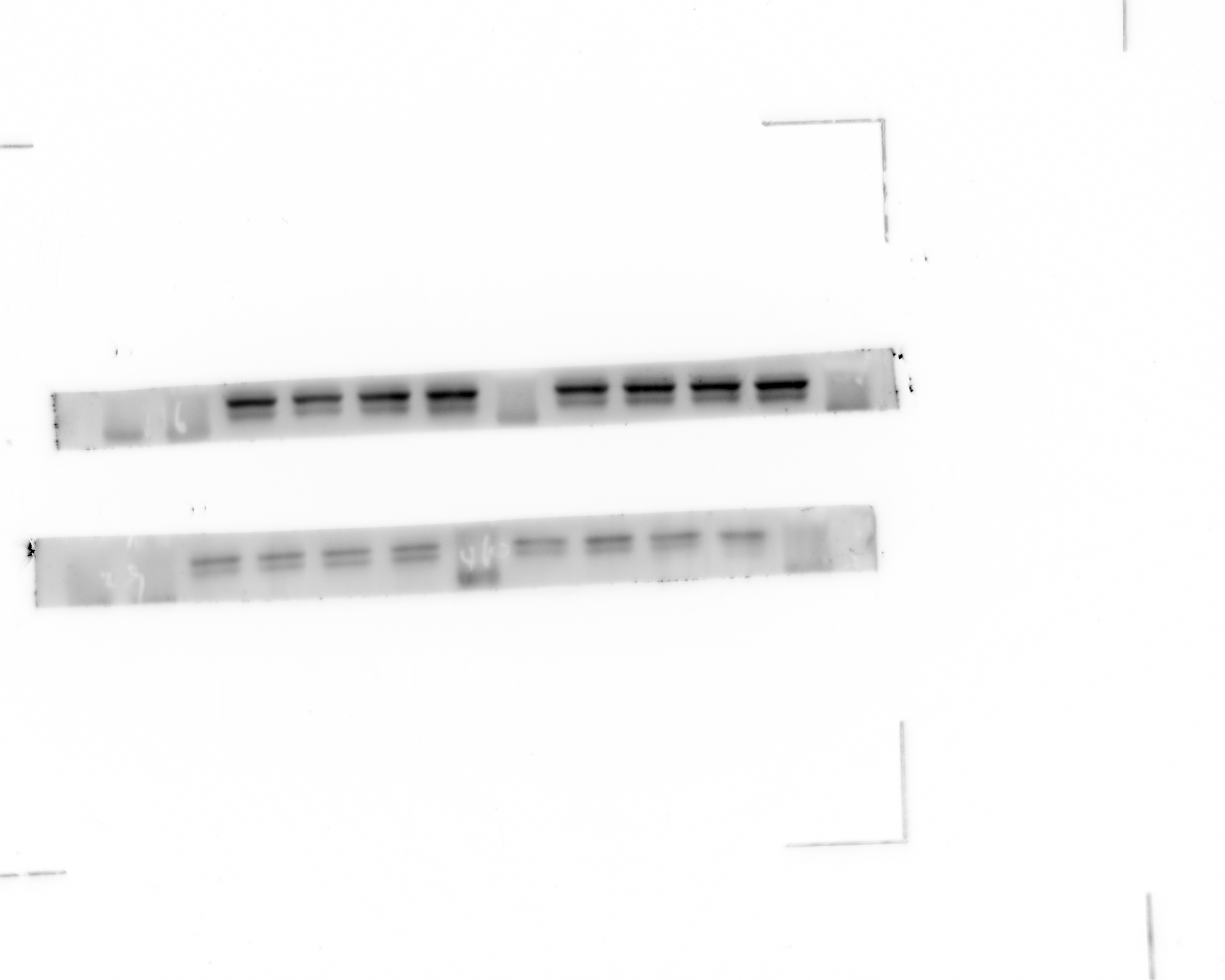


55KDa

**HCT 116+HT-29 mTOR (289 KDa):**


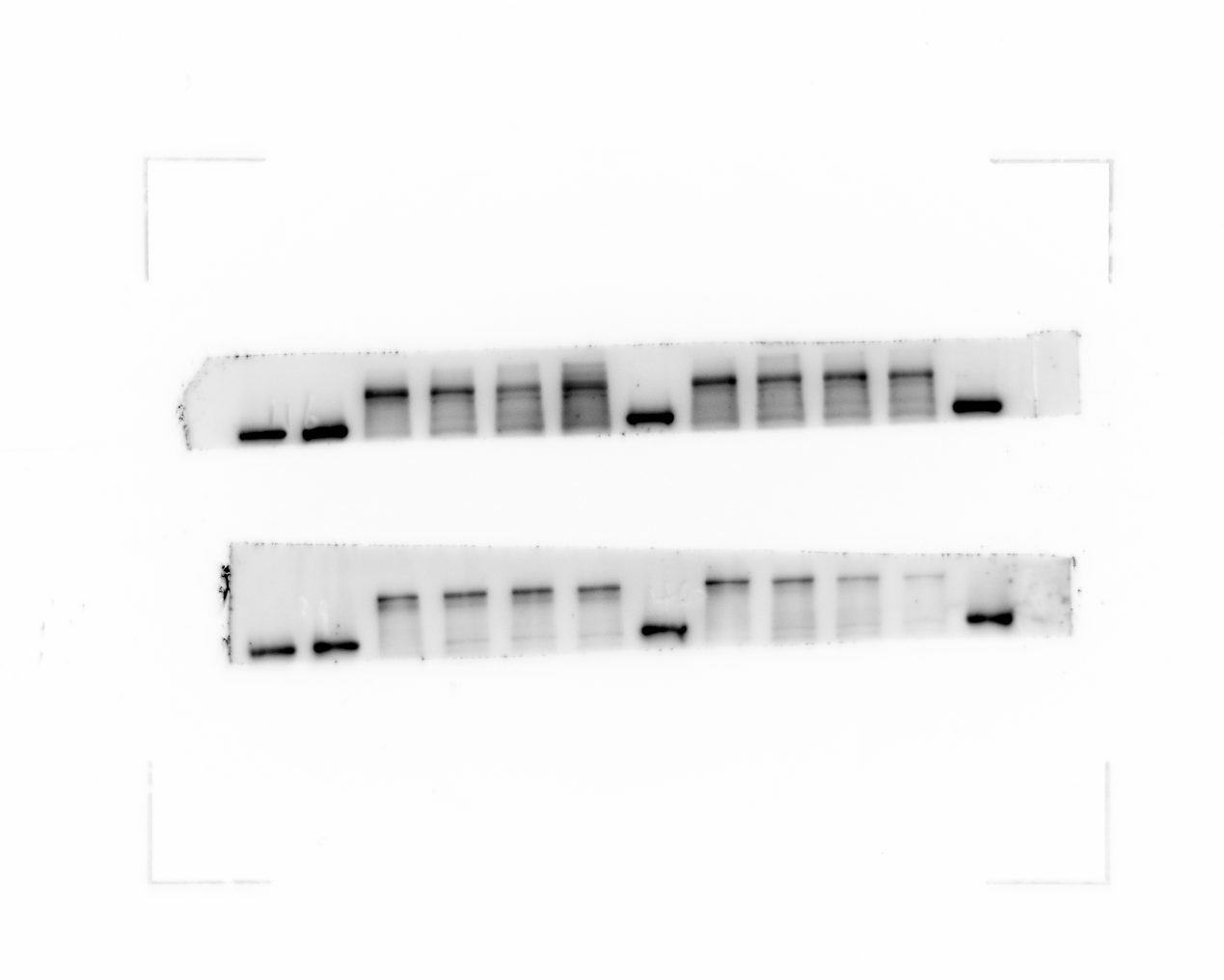


180 KDa

180 KDa

**NCM460 mTOR (289 KDa):**


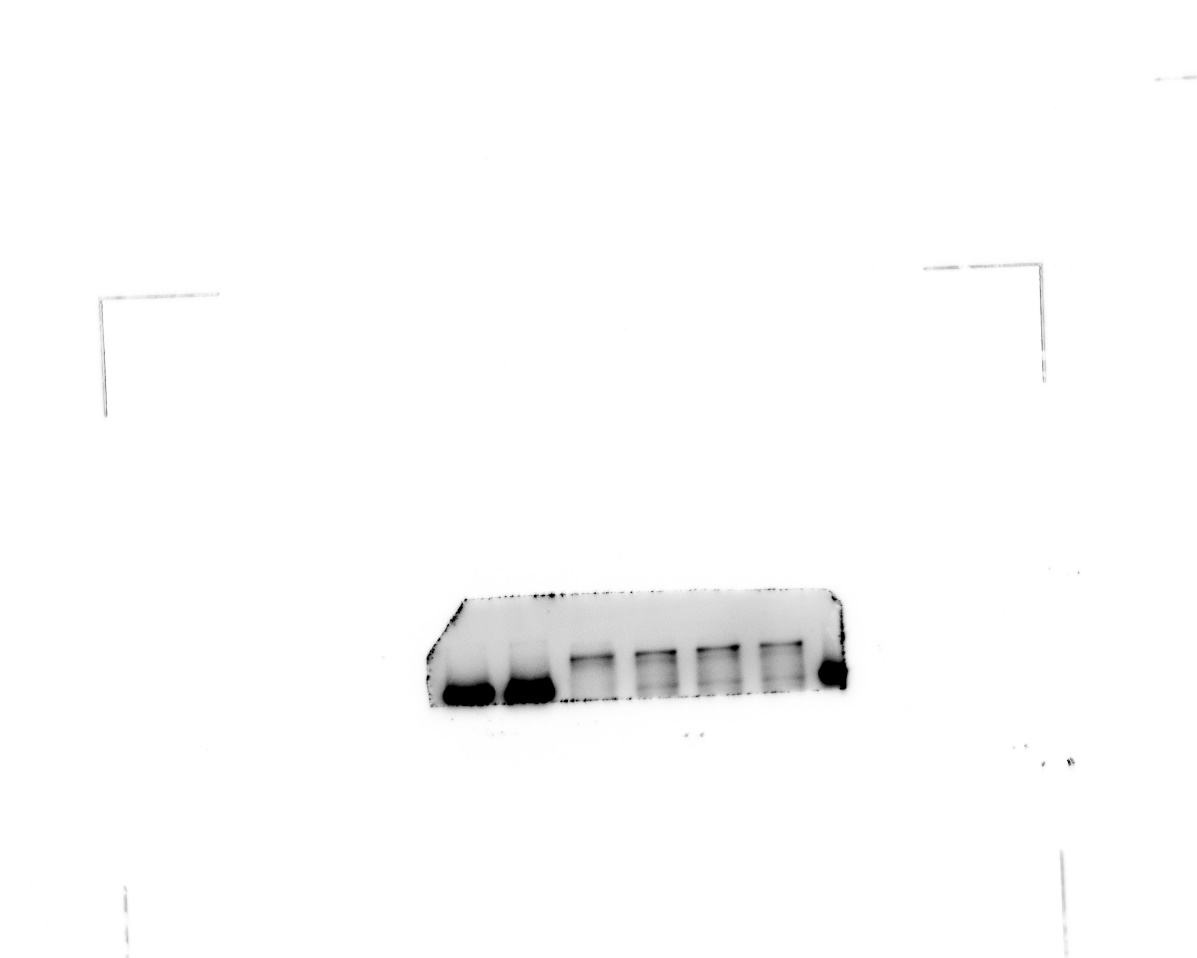


180 KDa

**HCT116 p-AKT (62 KDa):**


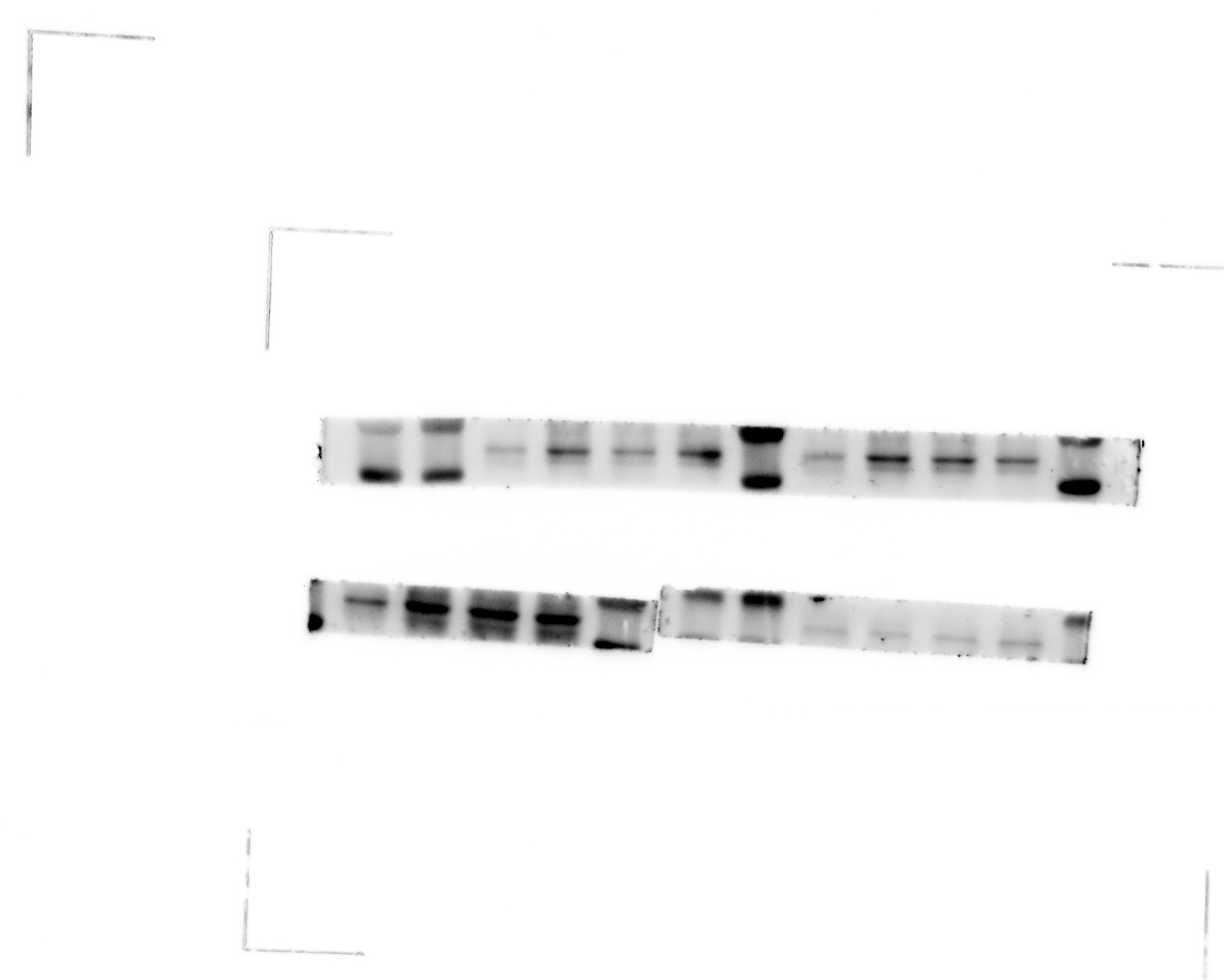


55 KDa

70 KDa

**NCM460+HT-29 p-AKT (62 KDa):**


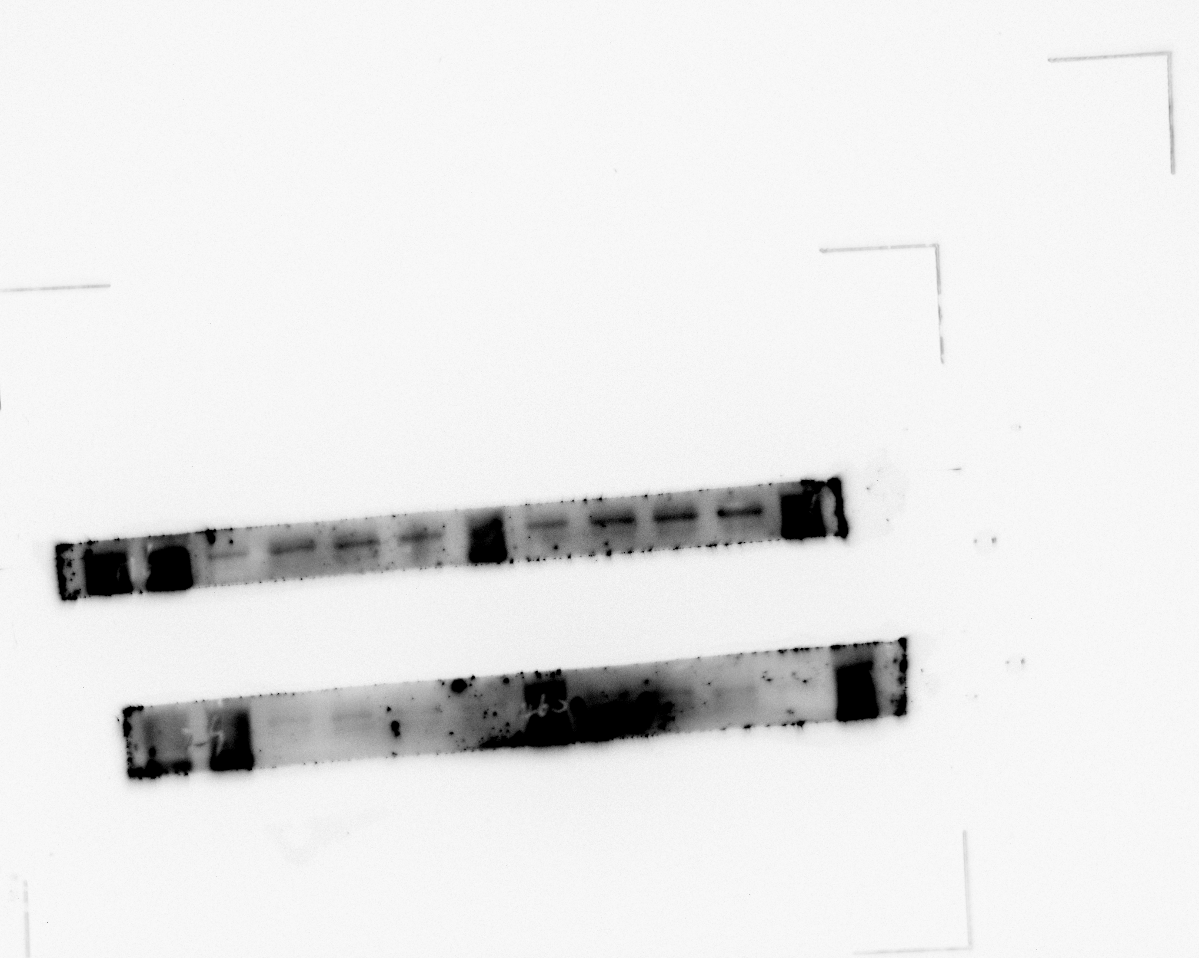


70 KDa

55 KDa

**HT-29 PI3K (85 KDa):**


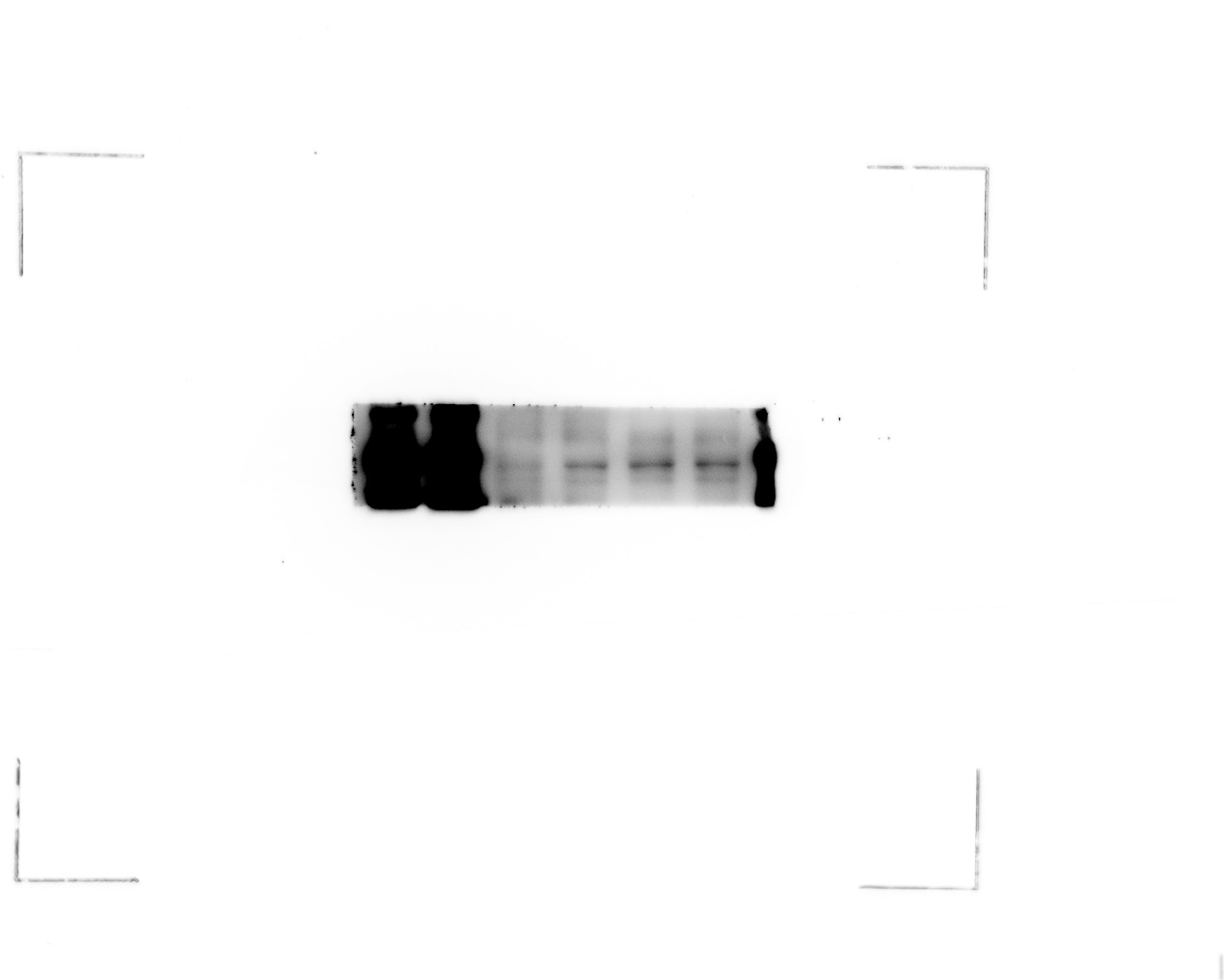


100 KDa

70 KDa

**HCT116 PI3K (85 KDa):**


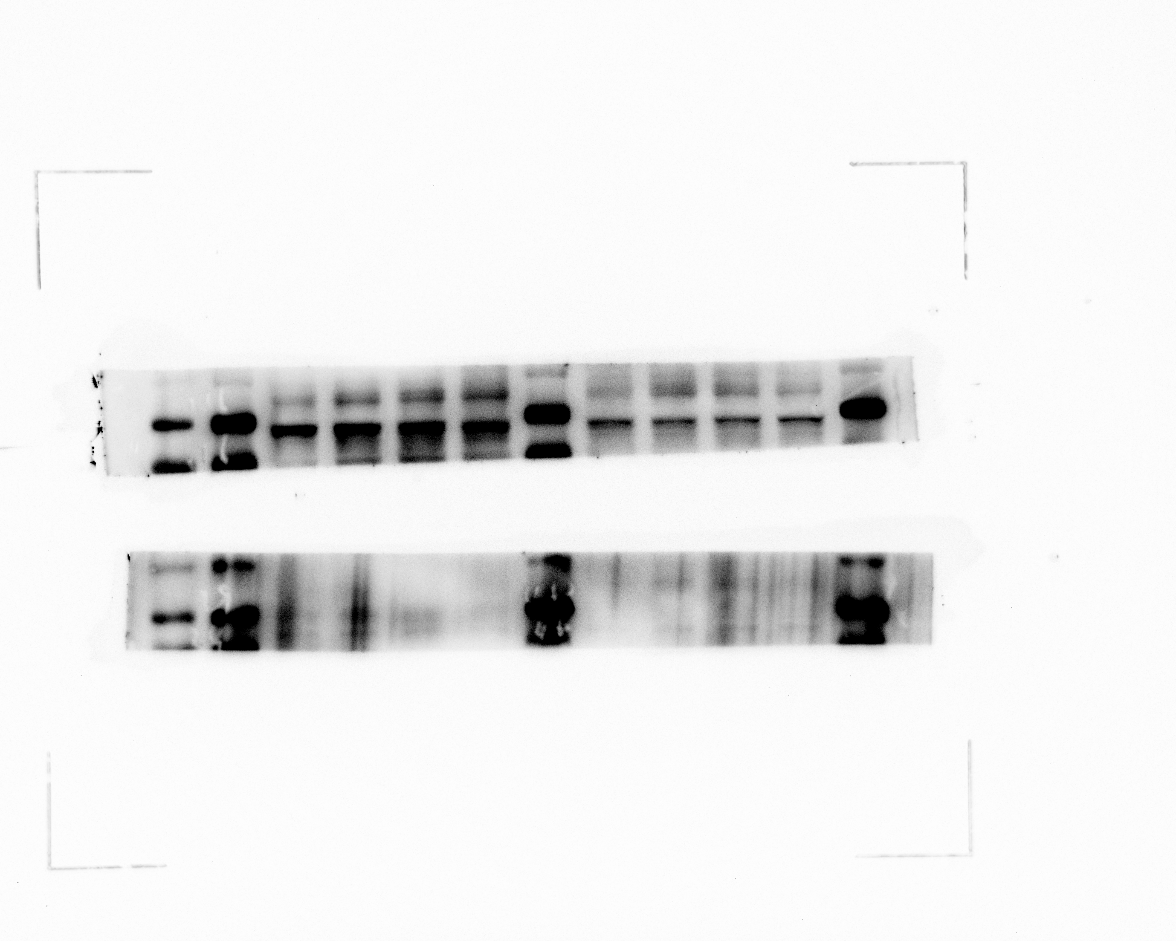


130 KDa

100 KDa

70 KDa

**NCM460 PI3K (85 KDa):**


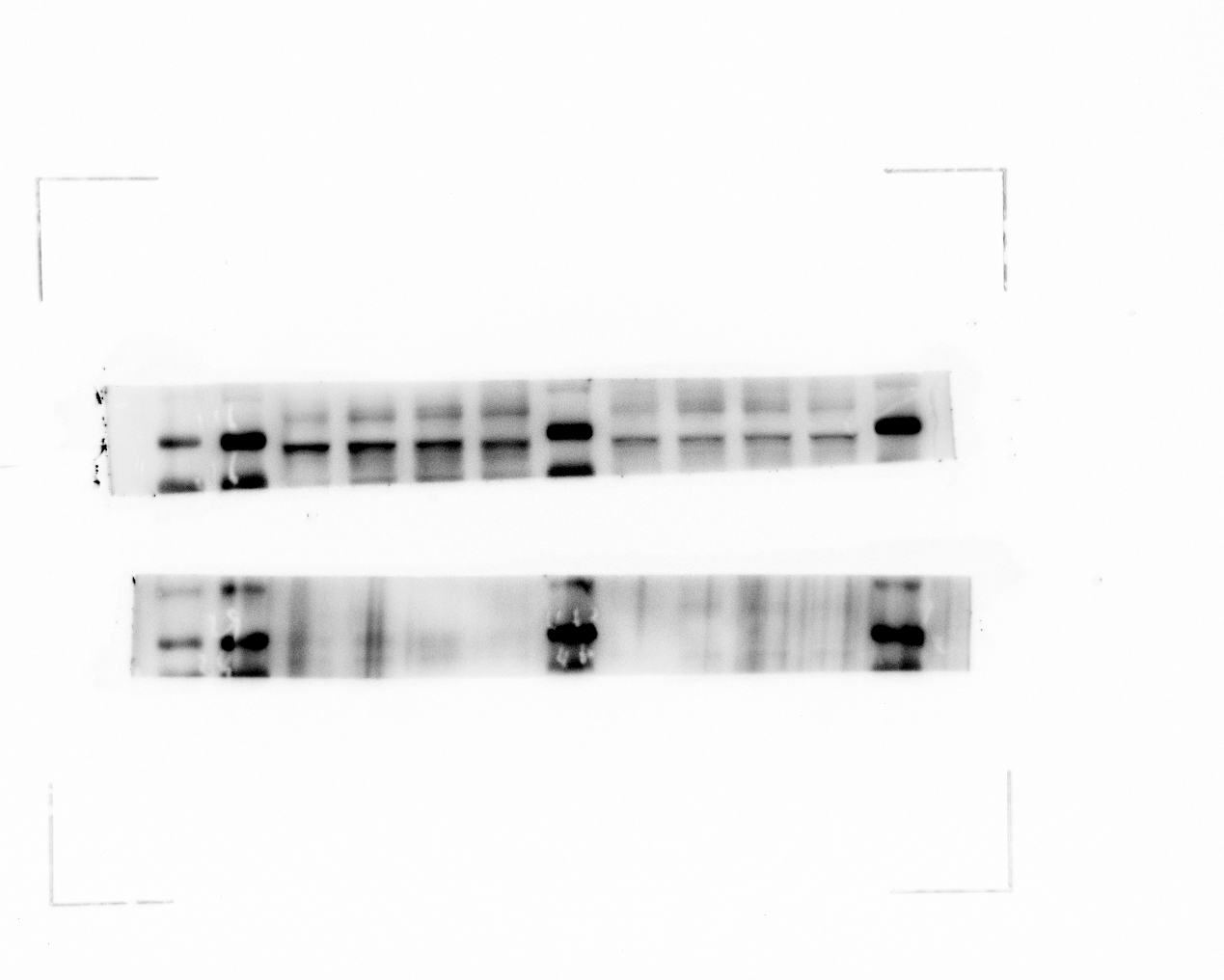


100 KDa

70 KDa

**NCM460 +HCT 116 p-mTOR (289 KDa):**


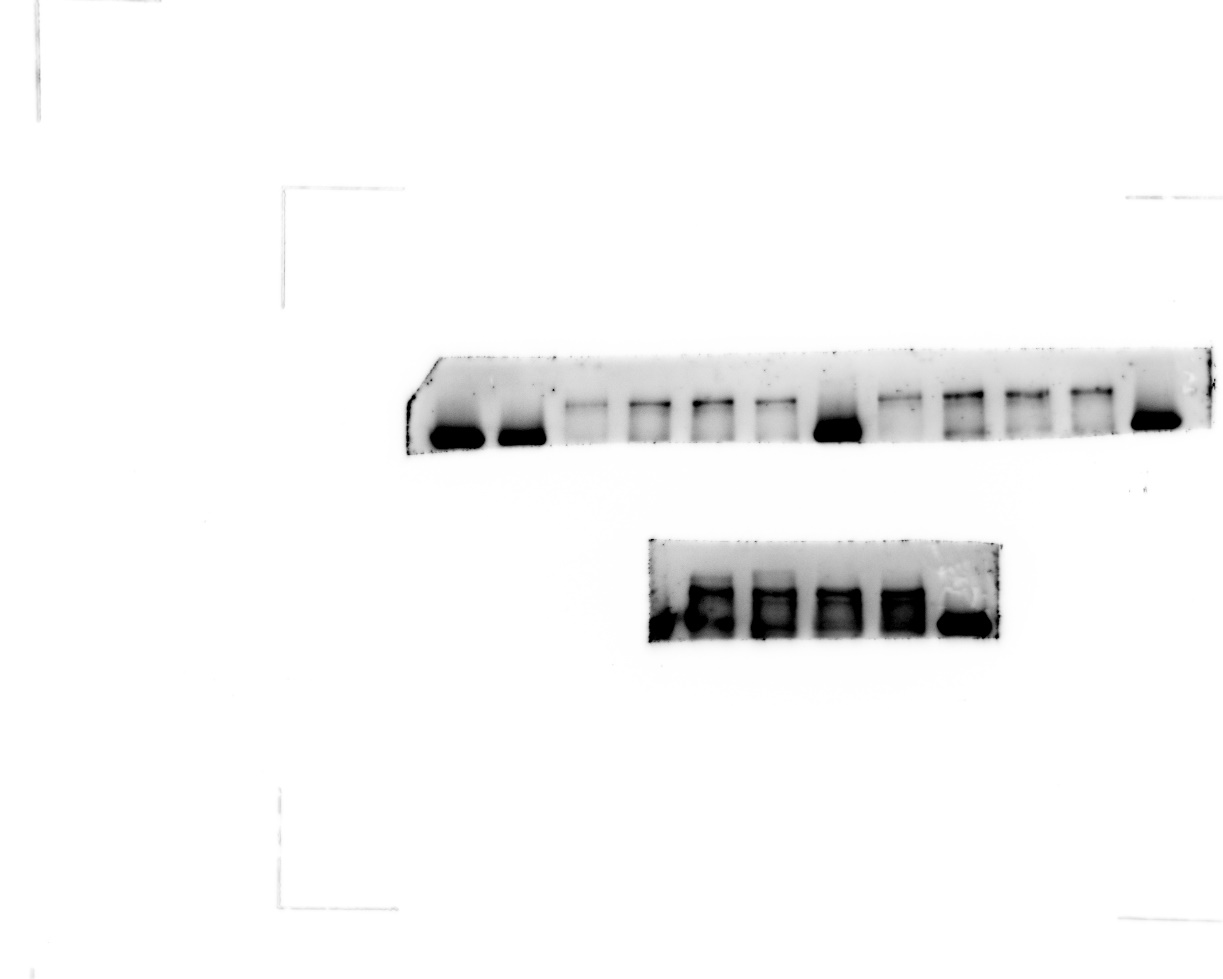


180 KDa

**HT-29 p-mTOR (289 KDa):**


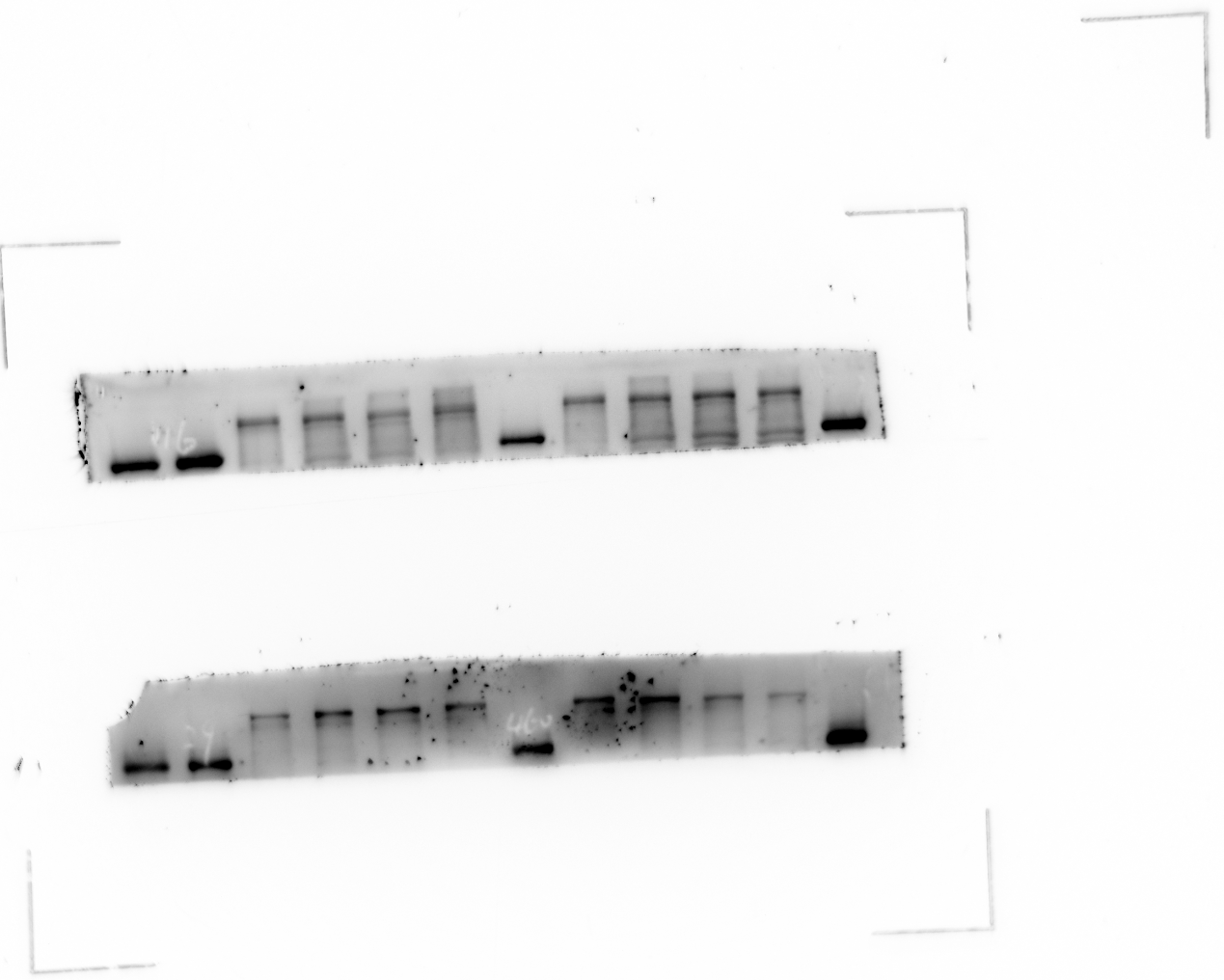


180 KDa

**p-PI3K (85 KDa):**


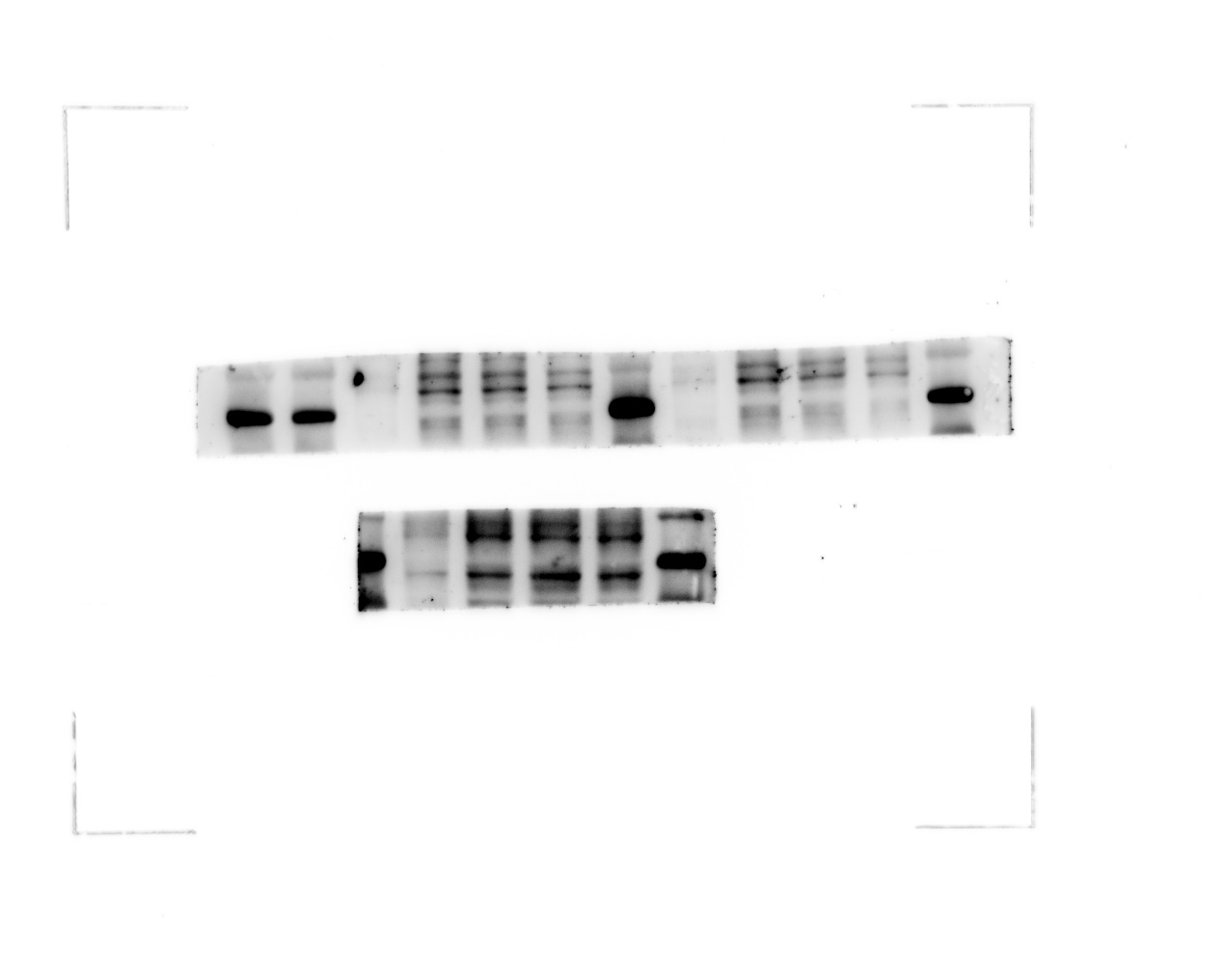


100 KDa

70 KDa

100 KDa

70 KDa

**Replicates Blots**

Actin-1:


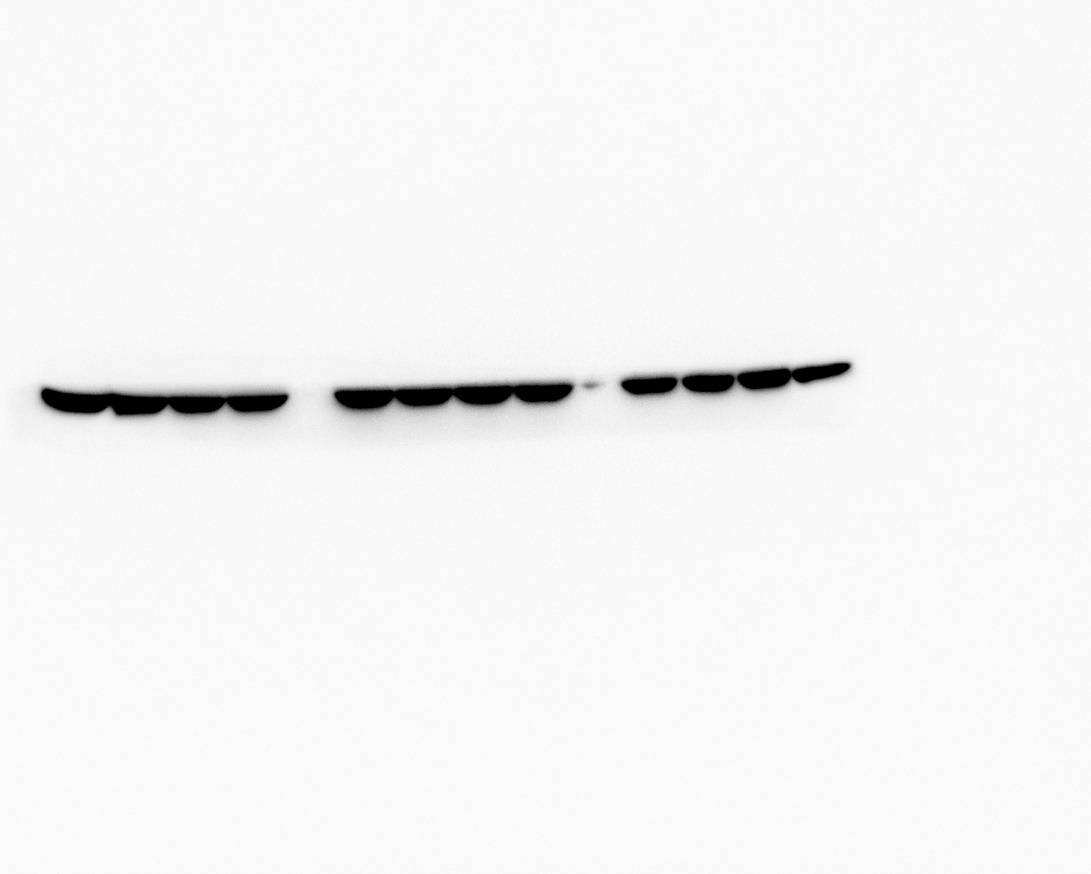


Actin-2:

**
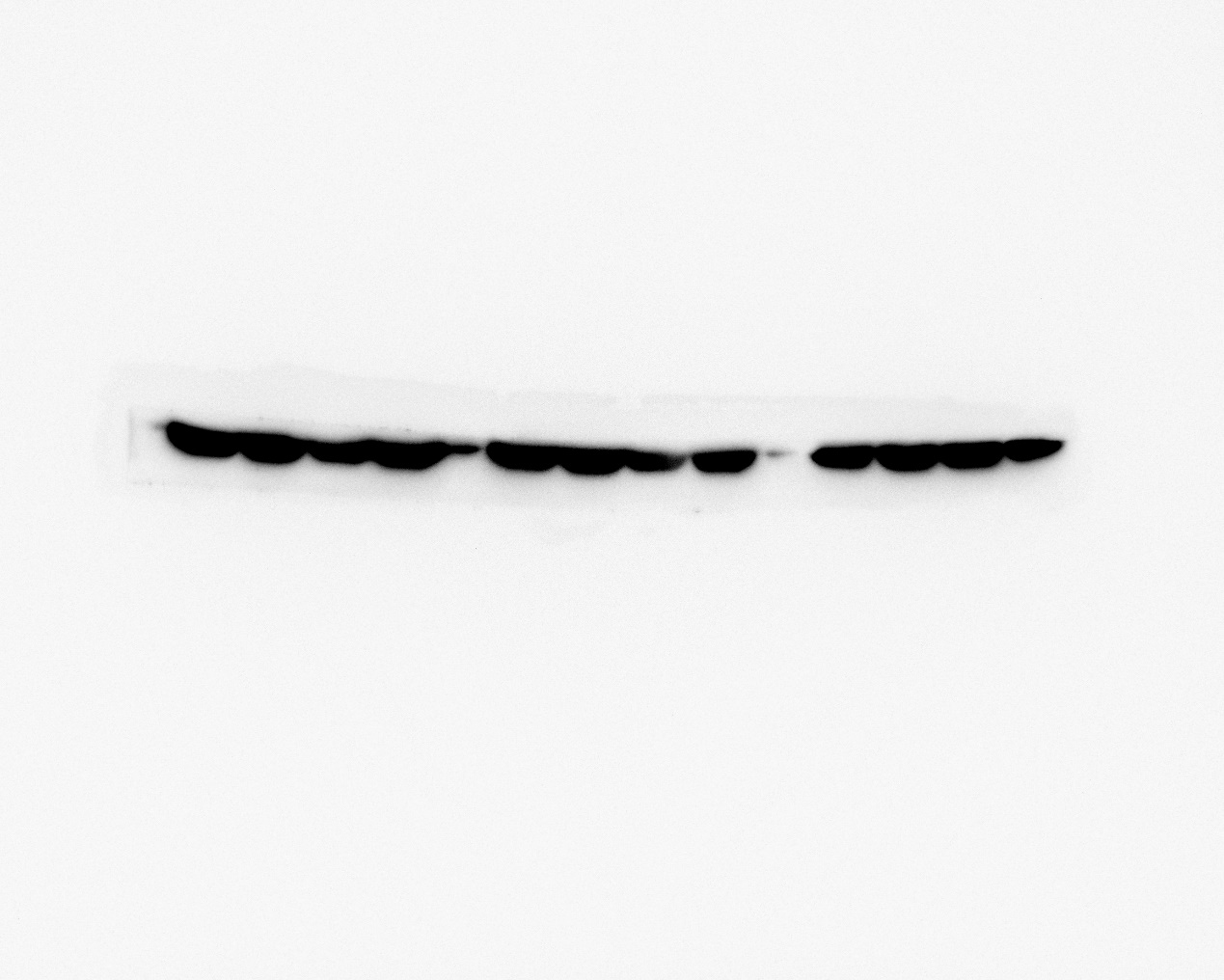
**

**PI3K-1：**

**
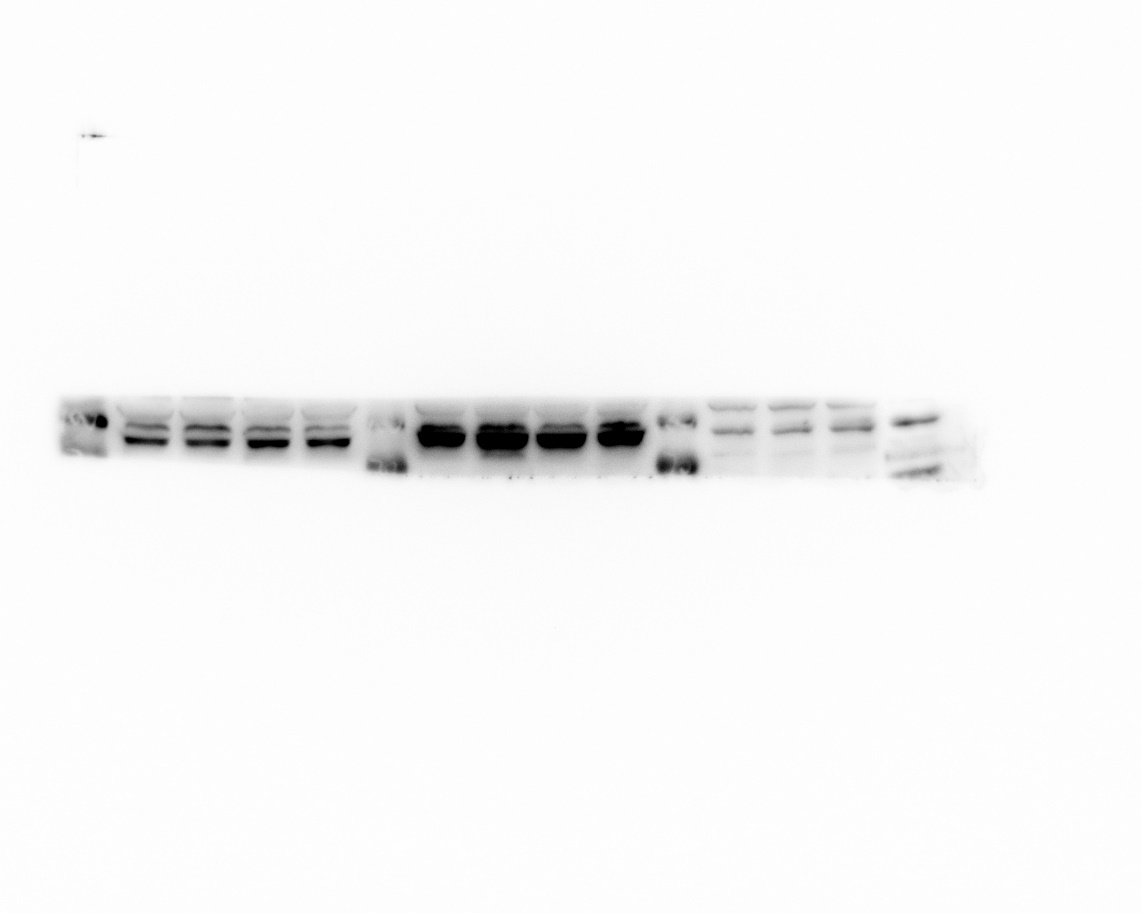
**

**PI3K-2：**

**
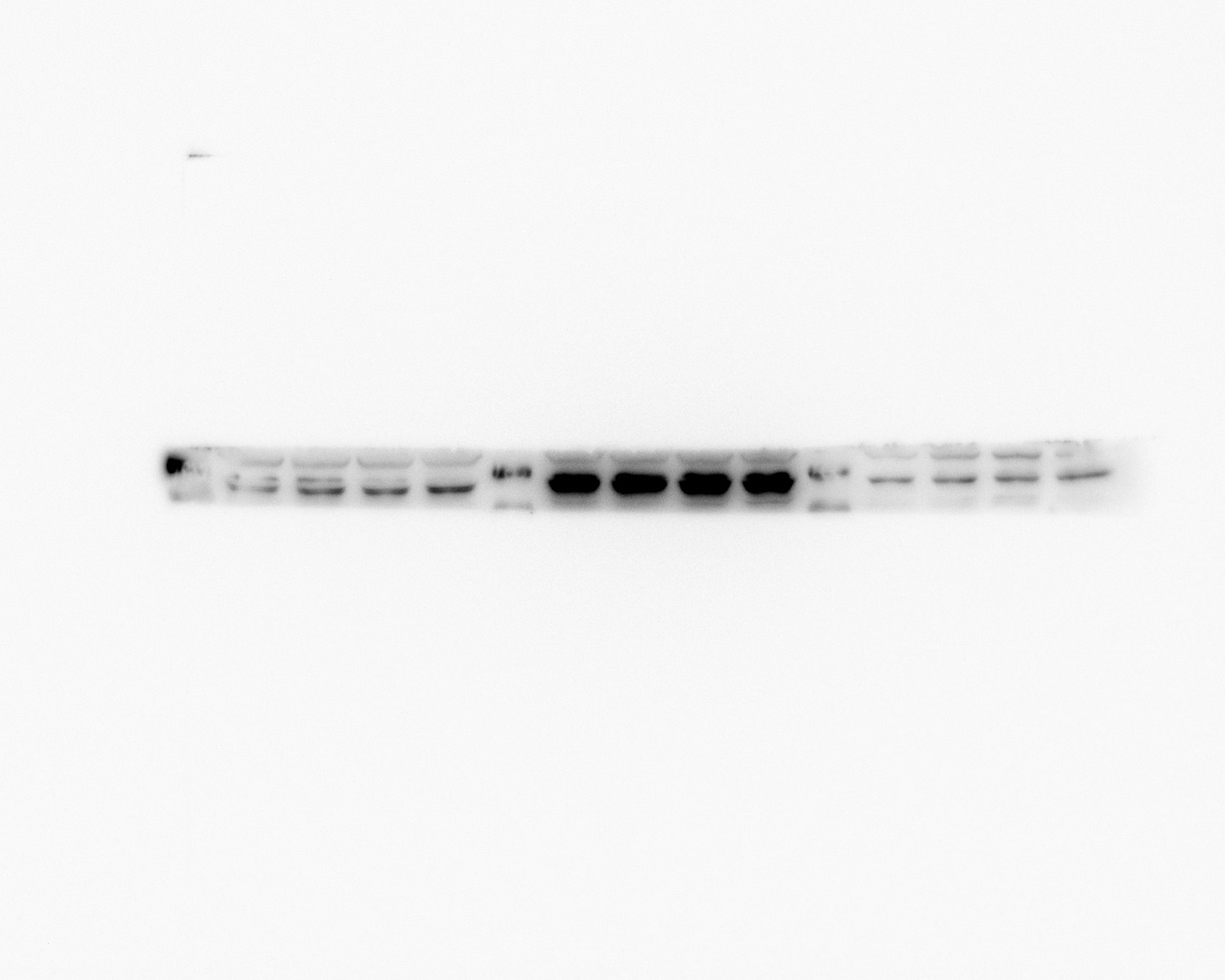
**

**AKT-1:**

**
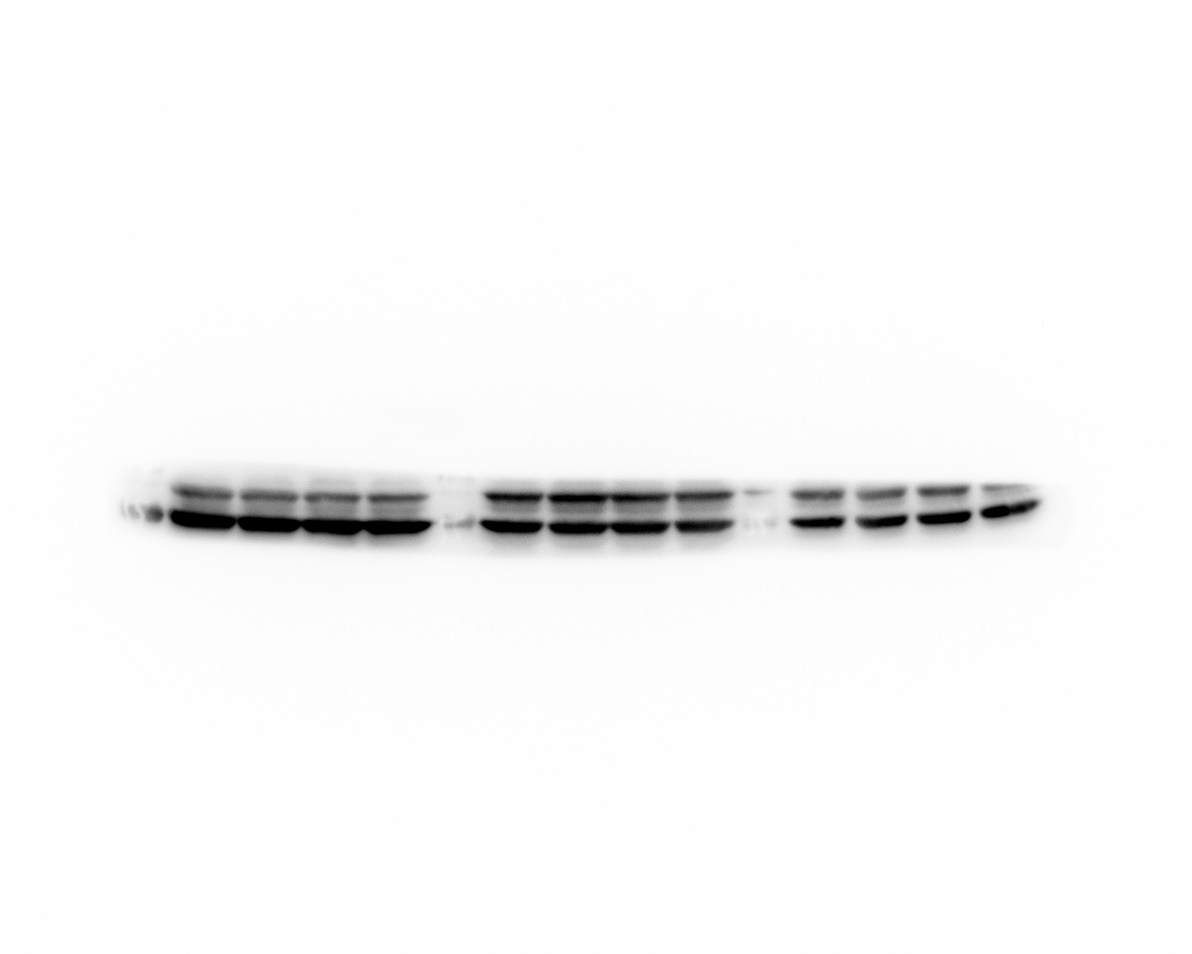
**

**AKT-2:**

**
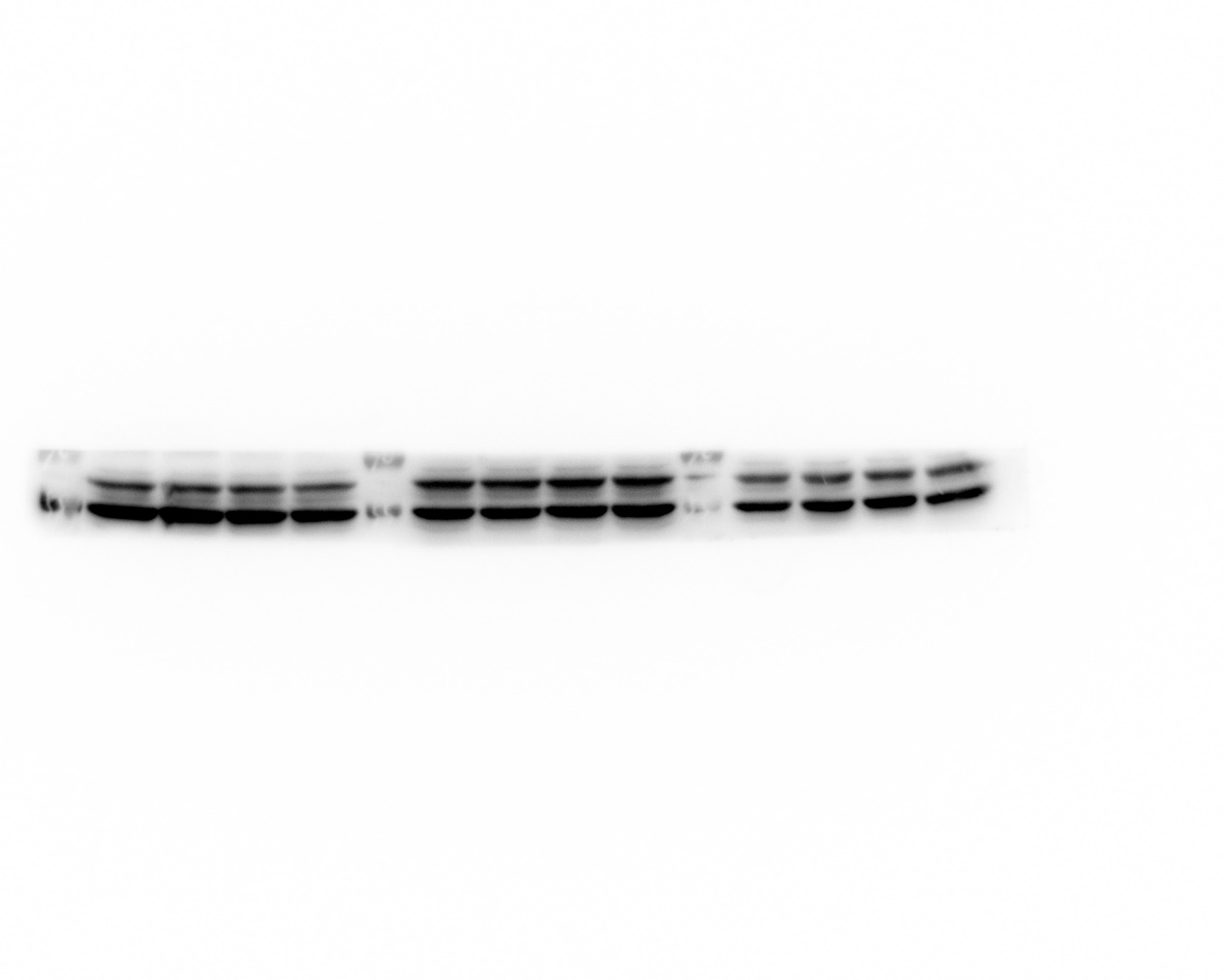
**

**mTOR-1:**

**
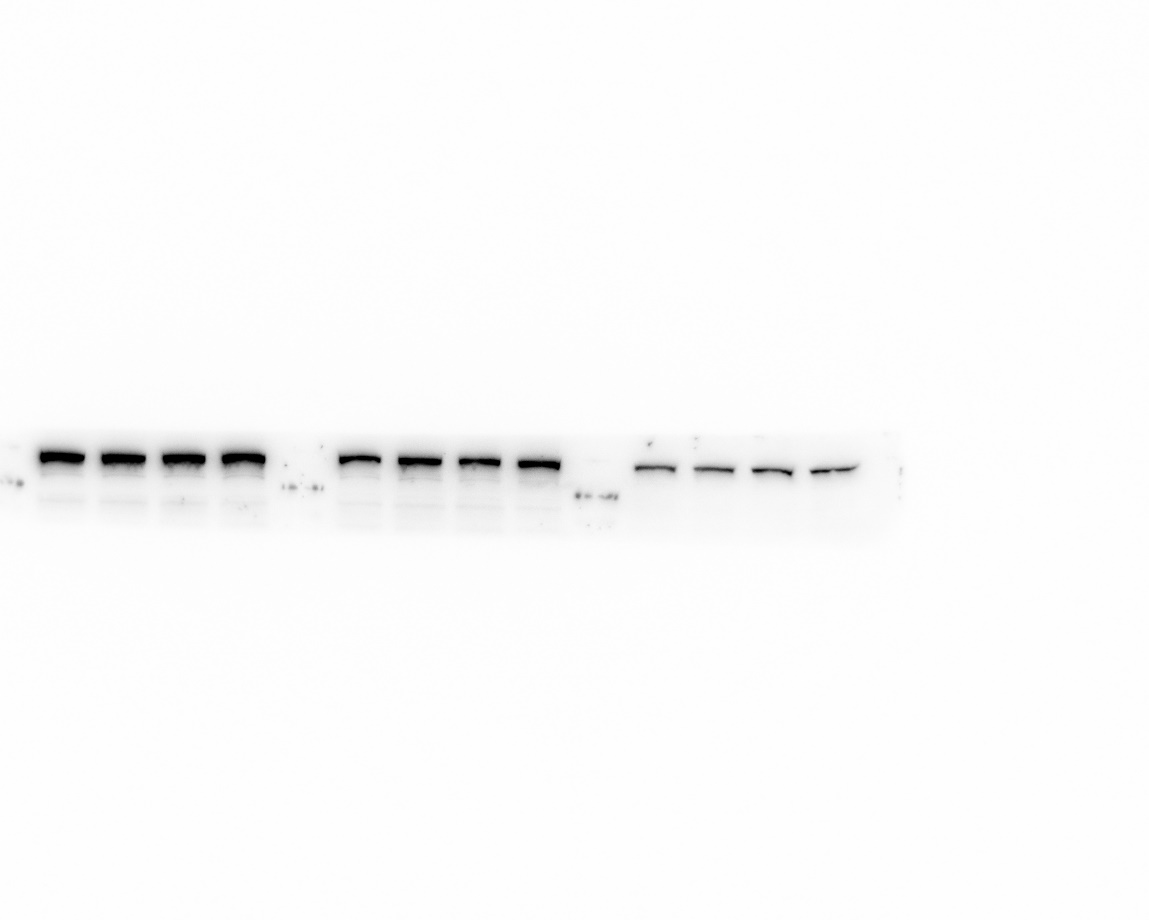
**

**mTOR-2:**

**
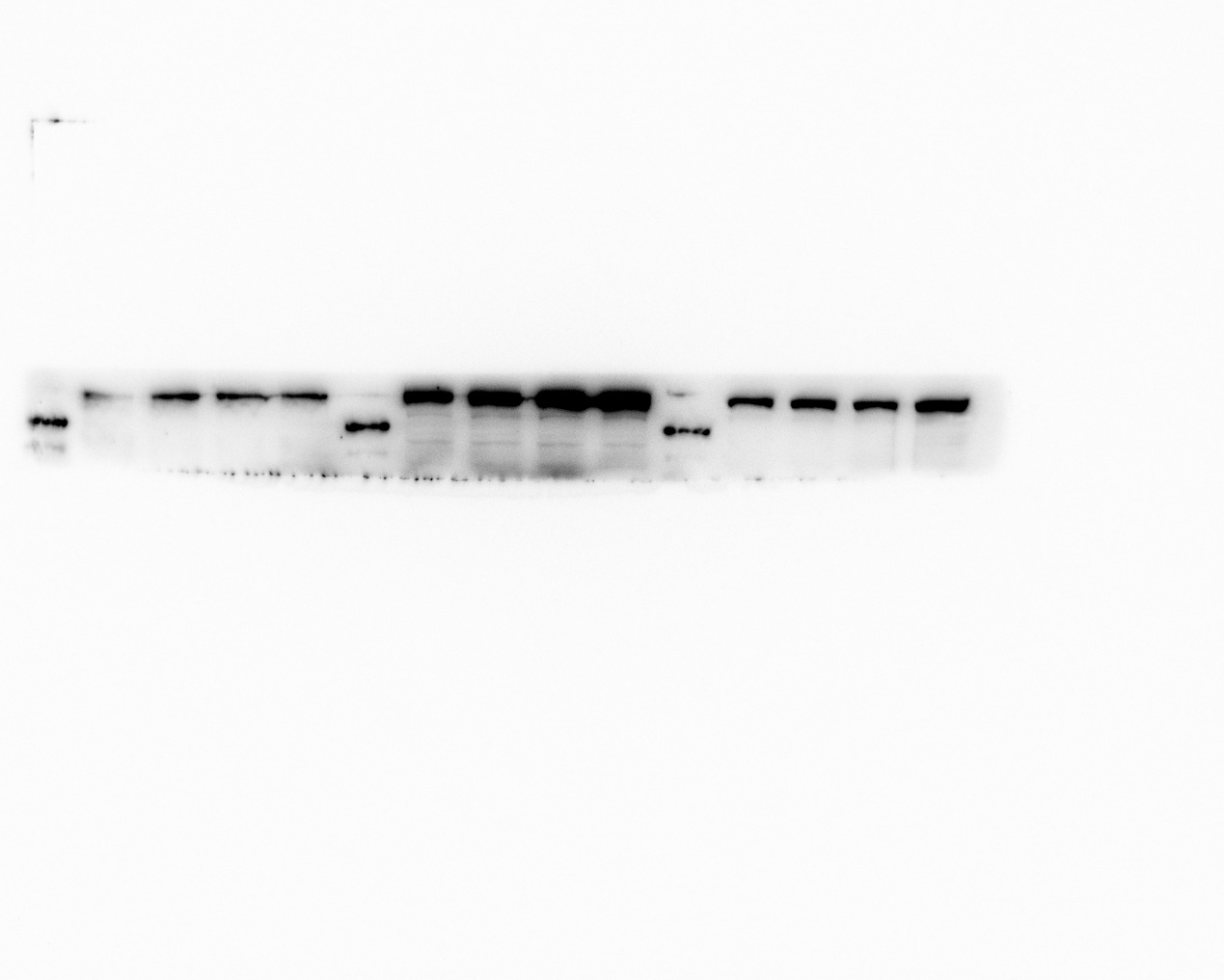
**

**p-PI3K-1:**

**
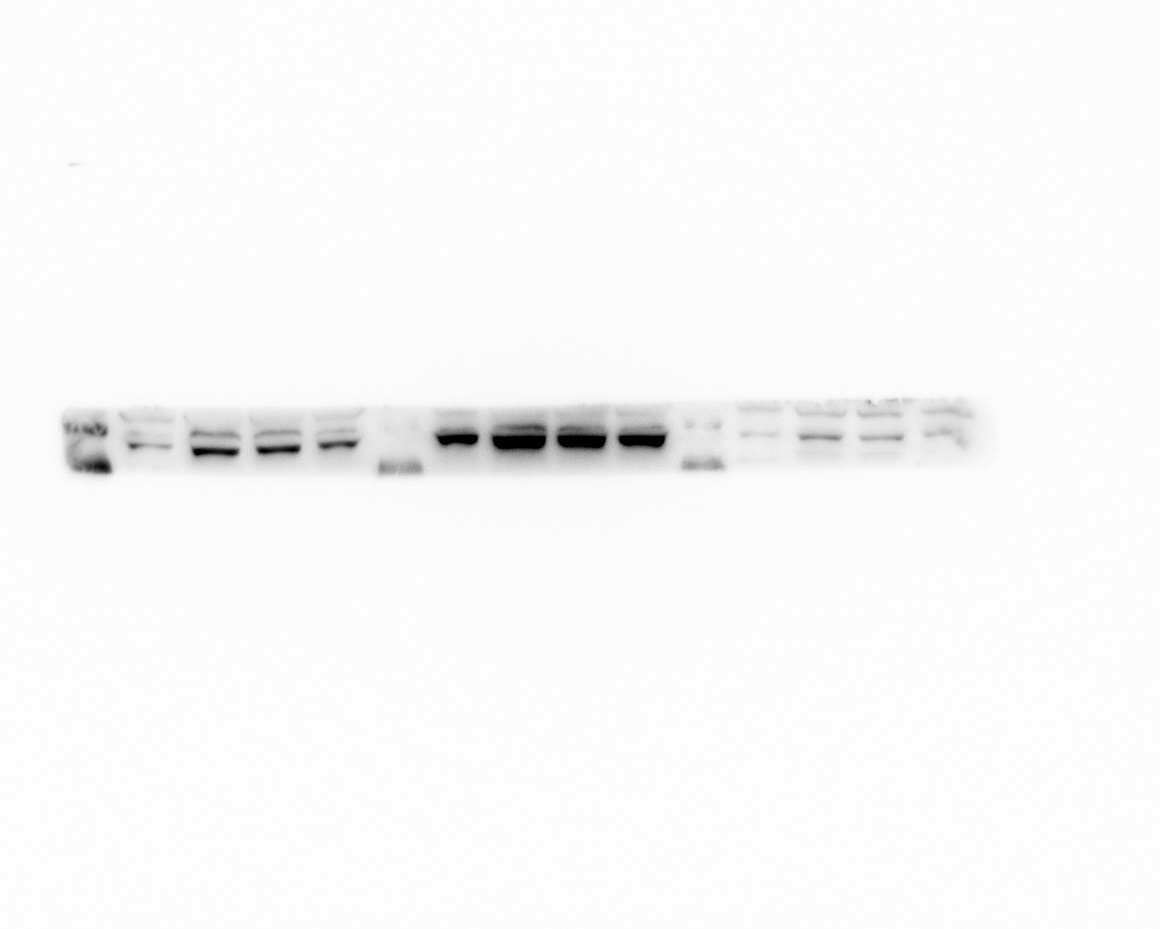
**

**p-PI3K-2:**

**
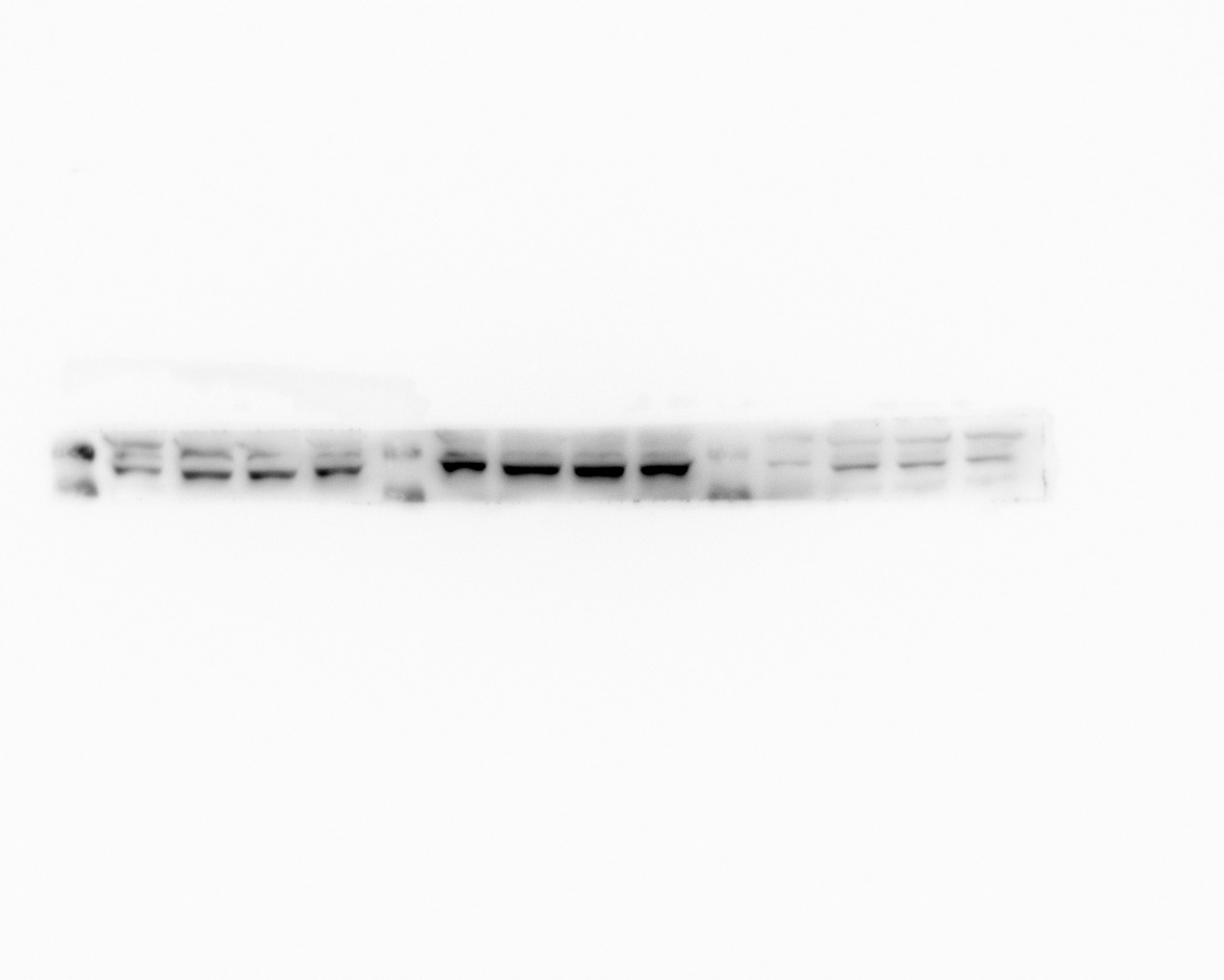
**

**p- mTOR -1:**

**
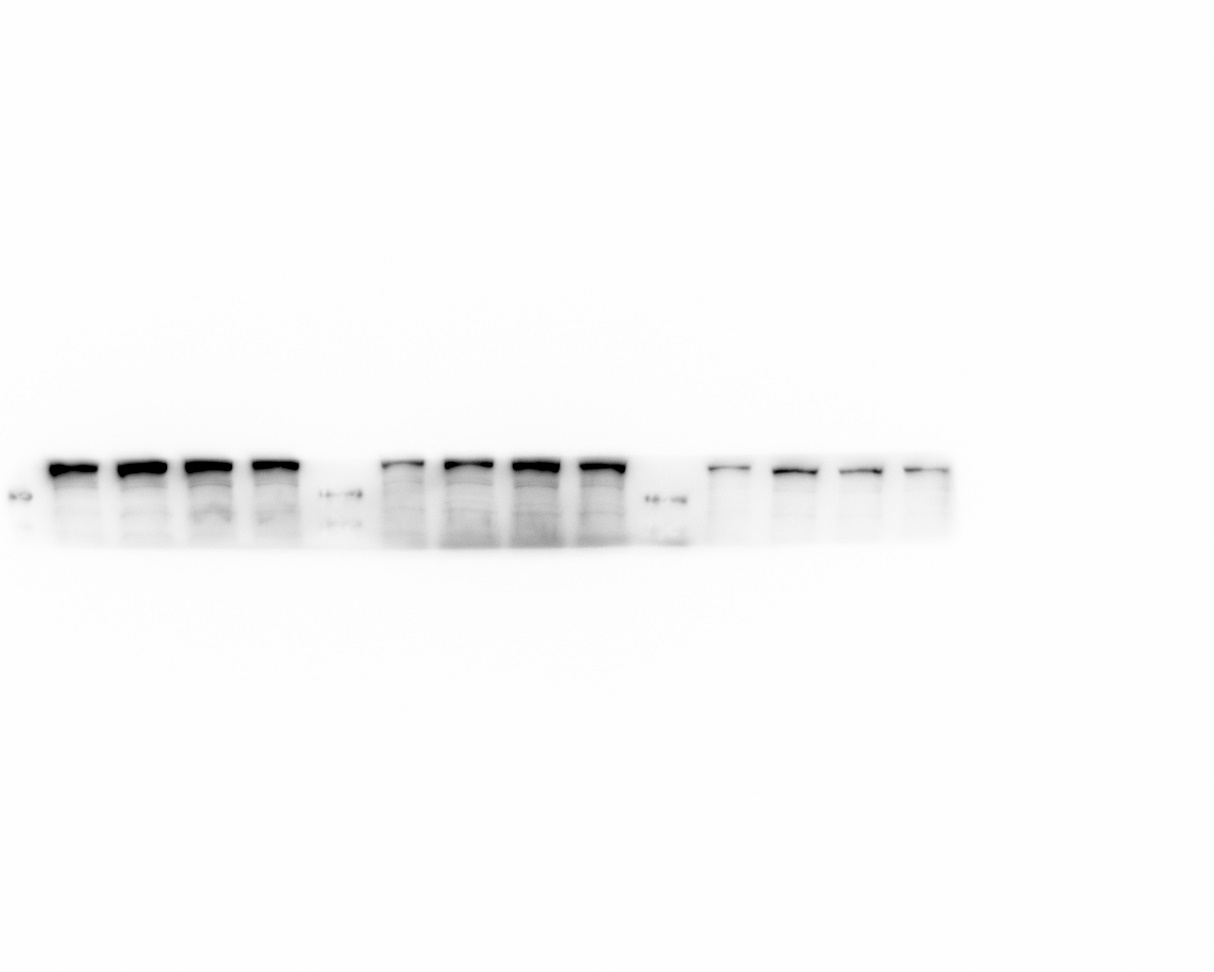
**

**p- mTOR -2:**

**
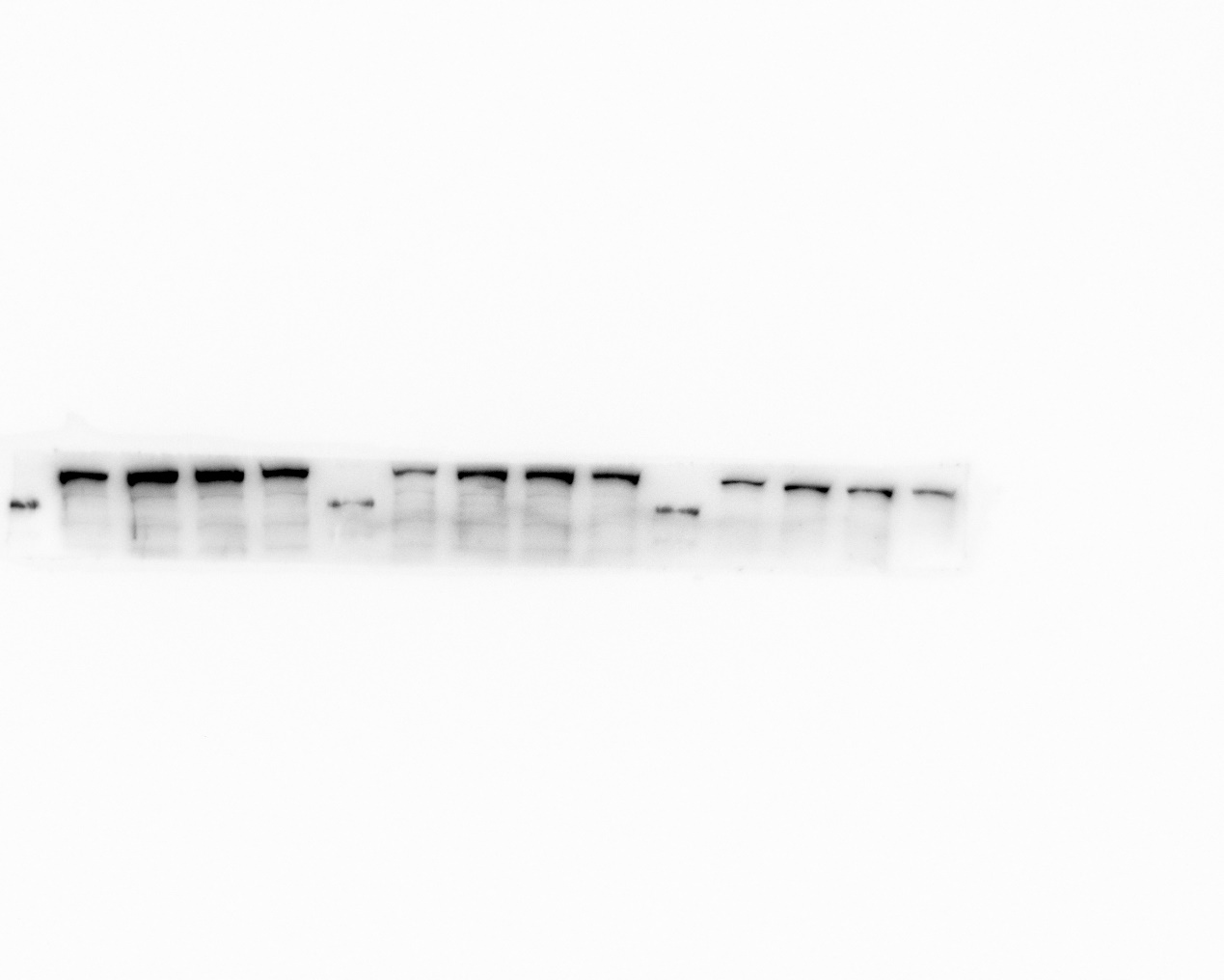
**
